# Circulating Fibroblast Growth Factor-21 in Patients with Nonalcoholic Fatty Liver Disease: A Systematic Review and Meta-Analysis

**DOI:** 10.1007/s13679-025-00643-x

**Published:** 2025-06-04

**Authors:** Ioanna Filimidou, Myrsini Orfanidou, Antonis Goulas, Olga Giouleme, Stergios Α. Polyzos

**Affiliations:** 1 First Laboratory of Pharmacology, School of Medicine, Aristotle University of Thessaloniki, Thessaloniki, Greece; 2 Second Propaedeutic Medical Department, School of Medicine, Hippokration General Hospital of Thessaloniki, Aristotle University of Thessaloniki, Thessaloniki, Greece

**Keywords:** Fibroblast growth factor-21, Metabolic dysfunction-associated steatohepatitis, Metabolic dysfunction-associated steatotic liver disease, Nonalcoholic fatty liver disease, Nonalcoholic steatohepatitis

## Abstract

**Background:**

The pathogenesis of nonalcoholic fatty liver disease (NAFLD) is multifactorial. Fibroblast growth factor-21 (FGF-21) has been proposed to be associated with NAFLD, but data on its circulating levels in patients with NAFLD are to date conflicting.

**Aims:**

The synthesis and comparison of data on circulating FGF-21 between patients with NAFLD and controls without NAFLD.

**Methods:**

A comprehensive literature search was conducted in PubMed, Cochrane Library and Scopus, complemented by hand-searching. Forty-four observational studies with overall 15,563 participants (9548 controls and 6015 NAFLD patients) were included in the study.

**Results:**

Circulating FGF-21 was higher in patients with NAFLD compared to controls (standardized mean difference [SMD]: 0.61; 95% confidence interval [CI]: 0.44, 0.77; p < 0.00001). Subgroup analysis showed higher FGF-21 levels in patients with nonalcoholic steatohepatitis (NASH) compared to controls (SMD: 1.30; 95% CI: 0.35, 2.24; p = 0.007), but not between hepatic steatosis and controls, or hepatic steatosis and NASH. Furthermore, the findings were more robust in the subgroup of studies with NASH-related cirrhosis than those without them (p = 0.0004). Sensitivity analysis further supported the findings. Heterogeneity was high in all comparisons. Meta-regression analyses showed that FGF-21 SMD between NAFLD patients and controls was positively associated with the rate of patients with type 2 diabetes mellitus per study, and this could explain 49.2% of the heterogeneity among studies.

**Conclusions:**

Circulating FGF-21 levels were higher in NAFLD patients than controls, which may be possibly attributed to those with advanced disease (NASH and related cirrhosis).

**Lay summary:**

Circulating fibroblast growth factor-21 levels were higher in patients with nonalcoholic fatty liver disease compared to controls. This is primarily attributed to the higher levels observed in patients with advanced disease (steatohepatitis and related cirrhosis).

**Supplementary Information:**

The online version contains supplementary material available at 10.1007/s13679-025-00643-x.

## Introduction

Nonalcoholic fatty liver disease (NAFLD) is a highly prevalent chronic liver disease affecting at least one third of the global population [1]. It was first described in 1980 and now is the second major cause leading to hepatic transplantation in the US, due to the end-stage liver disease and hepatocellular carcinoma [2]. The frequency of NAFLD increases in parallel with obesity, type 2 diabetes mellitus (T2DM) and other components of the metabolic syndrome. Histologically, NAFLD ranges from hepatic steatosis that may progress to nonalcoholic steatohepatitis (NASH), hepatic fibrosis and liver cirrhosis [3]. Regarding its pathogenesis, NAFLD is a multifactorial disease. Various genetic variants, environmental factors and epigenetic modifications contribute to its pathogenesis [4]. Dysregulation in metabolic homeostasis, insulin resistance (IR) and imbalance of various cytokines, adipokines, hepatokines and other mediators also play central role to the pathogenesis of NAFLD [5].

In 2019, a new nomenclature, metabolic (dysfunction)-associated fatty liver disease (MAFLD), was proposed in order to emphasize metabolic dysregulation in NAFLD [6]. In 2023, a multi-society Delphi consensus proposed the term metabolic dysfunction-associated steatotic liver disease (MASLD) in order to replace the stigmatizing term “fatty” [7]. According to the latter consensus, NASH was recommended to be renamed as metabolic dysfunction-associated steatohepatitis (MASH) [7]. Apart from changes in the nomenclature, MAFLD and MASLD emerged with new diagnostic criteria, which may reflect better the pathophysiology of the disease [6, 7].

**Fig. 1:**
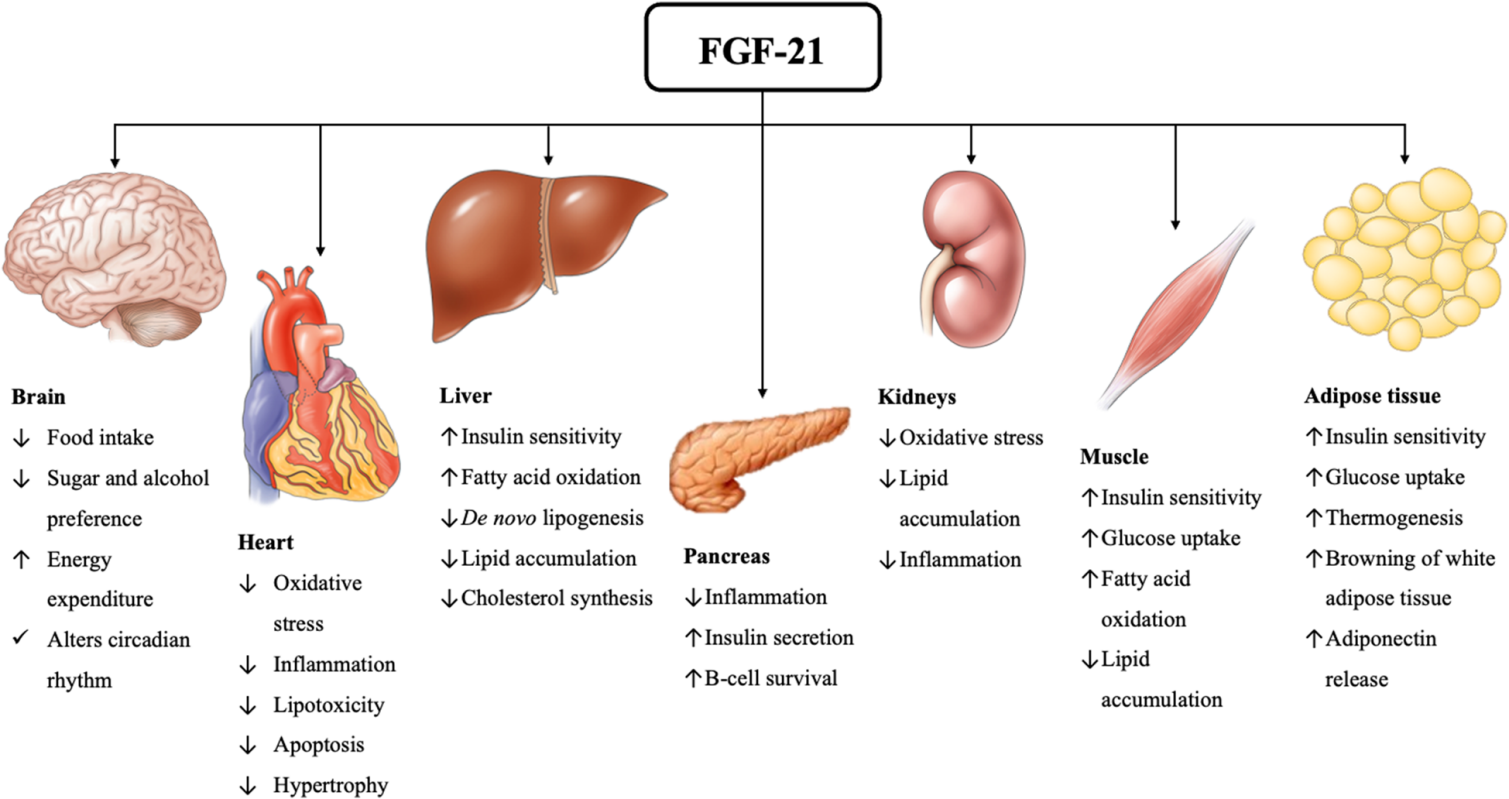
Potential metabolic effects of FGF-21 based mainly on experimental data [8–11]. FGF-21 is a hepatokine suggesting exerting pleiotropic effects on systemic metabolism by targeting a variety of organs through multiple endocrine pathways. These actions position FGF-21 as a potentially key metabolic regulator and a promising therapeutic target in obesity, T2DM and other metabolic diseases, including NAFLD. Abbreviations: FGF-21, fibroblast growth factor-21; NAFLD, nonalcoholic fatty liver disease; T2DM, type 2 diabetes mellitus

Fibroblast growth factor-21 (FGF-21) is an hepatokine with pleiotropic effects on lipid and carbohydrates metabolism [8]. FGF-21 was shown to decrease IR by augmenting the uptake of glucose in muscle; in the liver, FGF-21 decreases intrahepatic lipid accumulation by increasing fatty acid β-oxidation and decreasing de novo lipogenesis [8]. The potentially pleiotropic effects of FGF-21 on various organs, based mainly on data from experimental studies, are depicted in Fig. 1 [8–11]. In clinical terms, higher FGF-21 levels were shown in patients with NAFLD than in individuals without NAFLD in most, but not all studies; similarly, data on circulating FGF-21 levels between patients with simple hepatic steatosis and steatohepatitis are inconclusive. The clarification of FGF-21 levels when the disease advances seems to be important in the light of FGF-21 analogs that are currently under investigation in patients with NASH; higher FGF-21 levels may target to limit the progression of the disease, but may also indicate a state of FGF-21 resistance or insensitivity, which may complicate the effect of FGF-21 analogs [8].

Taking all the above into account, the main aim of this systematic review and meta-analysis was to quantitatively synthesize and compare existing data regarding circulating FGF-21 levels in patients with NAFLD and controls, i.e., individuals without NAFLD or other liver diseases. A secondary aim was to compare FGF-21 levels between patients with hepatic steatosis and NASH. In this systematic review and meta-analysis, we kept the terminology of NAFLD rather than the newer ones of MAFLD or MASLD, because the diagnosis of the diseases was based on the diagnostic criteria of NAFLD in the majority of the included studies.

## Methods

### Literature Search

This systematic review and meta-analysis was conducted based on a pre-registered protocol in the international prospective register of systematic reviews (PROSPERO) registry (CRD42024537642). The reporting guidelines of the meta-analysis of observational studies in epidemiology (MOOSE) was also followed for the preparation of this manuscript [12].

First, the following research question was developed based on the Population, Exposure, Comparison, Outcome PECO model; “Are FGF-21 levels higher in patients with NAFLD compared with individuals without NAFLD?”. Three databases, i.e., PubMed, the Cochrane Library and Scopus, were searched using a comprehensive search query. In order to build the search query, we combined Medical Subject Headings (MeSH) terms with non-MeSH terms and connected them with Boolean operators. The following query was used for the searching in the PubMed: (("Non-alcoholic Fatty Liver Disease"[Mesh]) OR NAFLD OR NAFL OR NASH OR (non-alcoholic fatty liver disease) OR (nonalcoholic fatty liver disease) OR (non alcoholic fatty liver disease) OR (non-alcoholic steatohepatitis) OR (nonalcoholic steatohepatitis) OR (non alcoholic steatohepatitis) OR (non-alcoholic fatty liver) OR (nonalcoholic fatty liver) OR (non alcoholic fatty liver) OR MAFLD OR (metabolic dysfunction-associated fatty liver disease) OR (metabolic dysfunction associated fatty liver disease) OR (metabolic associated fatty liver disease) OR MASLD OR (metabolic dysfunction-associated steatotic liver disease) OR MASH OR (metabolic dysfunction associated steatohepatitis)) AND (("fibroblast growth factor 21"[Supplementary Concept]) OR FGF-21 OR FGF21 OR (FGF 21) OR (Fibroblast Growth Factor 21) OR (Fibroblast Growth Factor-21)). There were no language or publication date restrictions. Small adjustments in the search string were made based on the specific requirements of each database. The search was performed independently by two investigators (IF and MO), starting in May 10, 2024 up to January 10, 2025.

The literature search was further expanded by manually search in reference lists of all articles included in the meta-analysis, as well as the abstract books of three major gastroenterology and hepatology conferences (the American Association for the Study of Liver Diseases, the European Association for the Study of the Liver and the Asian-Pacific Association for the Study of the Liver) between 2014 and 2024. Furthermore, automatic alerts were set up in the PubMed ("My NCBI"), the Cochrane Library ("Saved Search Alert") and Scopus ("Alerts"), to retrieve any relevant articles published after the initial search until the submission of this manuscript.

### Inclusion and Exclusion Criteria

This is a meta-analysis of observational studies. Therefore, cross-sectional, case–control and cohort studies providing data on circulating FGF-21 levels for individuals with and without NAFLD were eligible. Inclusion criteria were: (i) studies including patients diagnosed with NAFLD using: hepatic histology after liver biopsy, abdominal ultrasonography, transient elastography, computed tomography (CT), magnetic resonance imaging (MRI), MRI-proton density fat fraction (MRI-PDFF), magnetic resonance spectroscopy (MRS), magnetic resonance elastography (MRE), other relevant imaging techniques, or noninvasive markers of hepatic steatosis and/or fibrosis; studies following the nomenclature of MAFLD or MASLD were also eligible; (ii) studies reporting quantitative measurement of circulating FGF-21 levels in the plasma or serum.

Exclusion criteria were: (i) studies with patients with other liver disease (e.g., alcoholic fatty liver disease, viral hepatitis, autoimmune hepatitis, drug-induced liver injury) or with mixed liver diseases (patients with NAFLD with other concomitant liver diseases); (ii) overlap of patients in different studies; (iii) studies with patients with NAFLD-associated hepatocellular carcinoma; (iv) studies for which additional data (e.g., FGF-21 levels per group) were absolutely necessary, but the corresponding author(s) did not provide them; (v) other types of studies, including experimental studies, reviews, opinions, editorials, commentaries, guidelines, hypotheses, book chapters, case reports or letters-to-the-editor; though, research letters-to-the-editor, i.e., containing original data, were considered.

When a study included more than one control group, the control group with the greatest similarity to NAFLD group was selected for the statistical analysis; in this regard, priority was given to body mass index (BMI), i.e., when a lean and an obese control group were included, we selected the obese group, which is usually more similar to the BMI of NAFLD patients.

### Data Extraction

Two reviewers (IF and MO) independently performed the data extraction. Excel (Microsoft, Redmond, WA, USA) and EndNote (Clarivate Analytics, Philadelphia, PA, USA) were implemented in this procedure. The duplicates were removed and, subsequently, the two reviewers screened the titles and abstracts of all identified articles (stage of screening), excluding those that did not meet the prespecified inclusion and exclusion criteria. Next, the two reviewers independently evaluated the full-text articles, to select the appropriate ones for inclusion in the systematic review (stage of eligibility). For the automatic translation of articles published in non-English languages, Google Translate (https://translate.google.gr) was used as a supplementary tool; communication with the corresponding authors was also conducted to confirm the validity of the translation of essential data of their articles. Any disagreements between the reviewers were discussed with the involvement of the supervisor (SAP), until agreement was reached. The supervisor (SAP) guided the reviewers during the whole process and resolved any conflicts.

The next step was the extraction of relevant parameters from the included studies: (i) general features of the study (first author’s surname, country of origin, publication year, study design); (ii) specific populations (e.g., children/adolescent population, morbidly obese population subjected to bariatric surgery, inclusion of patients with liver cirrhosis); (iii) main characteristics per group (number of patients or controls, age, sex, BMI, waist circumference, rate of T2DM); (iv) method of diagnosis of liver disease (NAFLD/MAFLD/MASLD) and method for the measurement of FGF-21 levels; (v) histological system used for the grading and staging of the liver disease; (vi) IR calculated with homeostasis model assessment-IR (HOMA-IR) in mean ± standard deviation; (vii) FGF-21 and liver function tests [alanine aminotransferase (ALT), aspartate aminotransferase (AST) and gamma-glutamyl transferase (GGT)] in mean ± SD.

In case essential data were missing (e.g., FGF-21 levels, number of patients per group), they were asked from the respective corresponding authors. In studies that essential data were available only in graphs, we used the online tool Graphreader (https://graphreader.com) to retrieve their numerical values. When needed, standard formulas were used through the online tool Meta-Converter (https://meta-converter.com) to merge study groups and to transform numerical data expressed in other forms to mean and SD. When essential data of importance were not available and the corresponding author(s) did not provide them, the study was excluded.

### Quality Assessment

Newcastle–Ottawa scale (NOS; Ottawa Hospital Research Institute, Ottawa, ON, Canada) was the tool that was independently used by the two reviewers (IF and MO) for the quality assessment of included studies. The validity of each study was assessed within three domains: (i) the selection of the groups; (ii) the comparability of the groups; (iii) the assessment of the outcome. The scale of NOS ranges from 0 (very poor quality) to 9 (the highest quality). Any disagreement between the reviewers was resolved after discussion with the involvement of the supervisor (SAP).

### Outcomes

The standardized mean difference (SMD) with 95% Confidence Interval (95% CI) of circulating FGF-21 between patients with NAFLD and controls was the main outcome of this meta-analysis. Additionally, based on data retrieved from studies with histological grading and staging of NAFLD, secondary outcomes were the SMD of circulating FGF-21 between: (i) patients with simple hepatic steatosis (nonalcoholic fatty liver; NAFL) and controls; (ii) patients with NASH and controls, (iii) patients with NAFL and NASH.

### Statistical Analysis

The softwares Revman (Review Manager, Version 5.4, Cochrane Collaboration, London, UK) and R (R Studio, the R Foundation for statistical computing, Vienna, Austria) were use for the statistical analysis. The level of statistical significance was set at P < 0.05 in all tests (two-sided). I^2^ test was used for the evaluation of heterogeneity among studies. Given the expected heterogeneity among studies, the analysis was based on a random-effects inverse-variance model. Egger’s test and visual assessment of the funnel plot asymmetry were used for the evaluation of the probability of publication. Subgroup analyses were conducted to compare circulating FGF-21 in: (i) patients with NAFL vs. controls; (ii) patients with NASH vs. controls; (iii) patients with NAFL vs. patients with NASH; (iv) studies with vs. without biopsy-proven NAFLD; (v) studies with NASH-related cirrhosis vs. studies without NASH-related cirrhosis (fibrosis stage F4). Furthermore, sensitivity analyses were conducted after excluding studies with: (i) children/adolescent populations; (ii) morbidly obese populations undergoing bariatric surgery; (iii) NOS score < 7; (iv) outliers of FGF-21 SMD; (v) the use of the definition of MAFLD for the diagnosis of the disease. Finally, meta-regression analysis with a random-effect model was conducted, to regress FGF-21 SMD between patients with NAFLD and controls for the following potential confounders: (i) age; (ii) sex; (iii) T2DM; (iv) BMI; (v) waist circumference; (vi) HOMA-IR.

## Results

### Literature Search

**Fig. 2:**
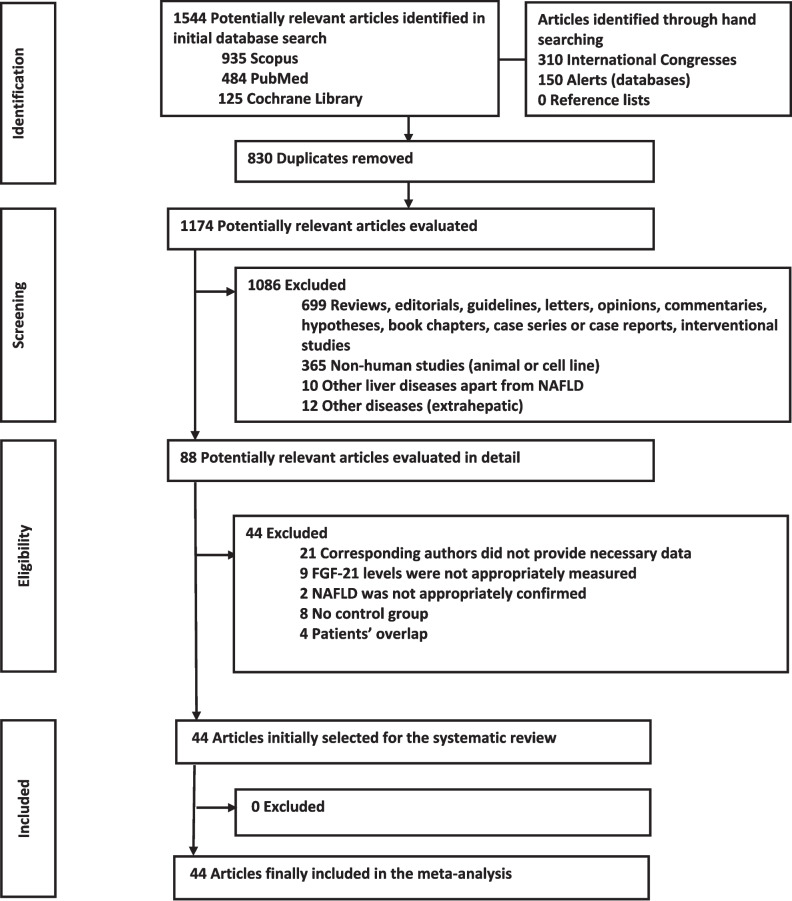
Flowchart depicting the process of the literature search, according to the PRISMA statement. Abbreviations: FGF-21, fibroblast growth factor-21; NAFLD, nonalcoholic fatty liver disease; PRISMA, preferred reporting items for systematic reviews and meta-analyses

The initial search led to the retrieval of 1544 articles: 935 from Scopus, 484 from Pubmed and 125 from Cochrane library. In addition, 150 articles were retrieved via automatic alerts set in the above databases and 310 articles through handsearching in the abstract books of the three international conferences mentioned above. The process of identification, screening, eligibility and of the final selection of studies to be included in the systematic review and meta-analysis is fully presented in Fig. 2, which follows the reporting guidelines of the Preferred Reporting Items for Systematic reviews and Meta-Analyses (PRISMA), and is briefly reported hereby. After the removal of 830 duplicates, 1086 articles were excluded at the stage of screening. Therefore, 88 articles were included in the stage of eligibility. Communication with the corresponding author(s) was required for 35 of these articles. Seventeen authors of 11 studies responded and provided us the required data; their fine contribution is recognized in the acknowledgement section of this article. On the contrary, the corresponding authors of 21 studies did not reply or were unwilling to provide critical information (e.g., FGF-21 levels) for their studies, which, therefore, were excluded. The authors of other three studies did not respond to less crucial queries (e.g., the method of FGF-21 measurement), so their studies were included in the meta-analysis. Finaly, 44 studies were included in this systematic review and meta-analysis [13–56].

### Descriptives of the Included Studies

The main descriptives of the 44 included studies are presented in Table 1. They were conducted between 2010 and 2024 and they totally contain data from 15,563 individuals, 9548 controls and 6015 patients. Eighteen studies were conducted in Europe, 19 in Asia, six in America and one in Africa. There were 36 cross-sectional studies, three case–control studies and five cohort studies, from which only the baseline data were used and analyzed in the meta-analysis. NAFLD was diagnosed with liver biopsy in 15 studies, abdominal ultrasonography in 14 studies, transient elastography in three studies, MRI-PDFF in three, MRI in two and MRS in two studies, CT in one, non-invasive indices in one and both ultrasonography and non-invasive indices in one study; the criteria of MAFLD were implemented in two studies, whereas those of MASLD in none. Furthermore, nine studies referred to pediatric/adolescent populations, four studies to patients with morbid obesity subjected to bariatric surgery (Table 1) and four studies included patients with NASH-related cirrhosis (Table S1). Circulating levels of FGF-21 were measured by enzyme-linked immunosorbent assay (ELISA) in 42 studies, except one that were measured with proximity extension immunoassay (PEI) and one that the method of FGF-21 measurement was not available (Table 1).

**Table 1:** Main descriptives of the studies included in the systematic review and meta-analysis

First author, Year, Origin^†^	Study design	Method of NAFLD diagnosis	Method of FGF-21 measurement	NOS score	Additional information
Abozaid, 2023, Netherlands [13]	Cohort study	Abdominal ultrasonography	PEI	8	
Ajaz, 2021, United Kingdom [14]	Cross-sectional	Liver biopsy	ELISA	4	All patients, but not controls, with NASH
Alisi, 2013, Italy [25]	Cross sectional	Liver biopsy	ELISA	8	Pediatric population
Babak, 2017, Ukraine [36]	Cross-sectional	Abdominal ultrasonography	ELISA	5	
Bahijri, 2023, Saudi Arabia [47]	Cross-sectional	Abdominal ultrasonography	ELISA	9	All patients and controls with T2DM
Barb, 2019, USA [52]	Cross-sectional	Liver biopsy	ELISA	8	
Chang, 2022, South Korea [53]	Cross-sectional	MRI -PDFF	ELISA	8	
Dushay, 2010, USA [54]	Cross-sectional	Liver biopsy	ELISA	7	
Elshinshawy, 2023, Egypt [55]	Cross-sectional	Transient elastography	ELISA	6	All patients, but not controls, with hypothyroidism; all patients and controls without T2DM
Flisiak-Jackiewicz, 2019, Poland [56]	Cross-sectional	Abdominal ultrasonography	ELISA	7	Pediatric population; all patients and controls without T2DM
Franck, 2023, Germany [15]	Cross sectional	Liver biopsy	ELISA	5	
Gallego-Duran, 2024, Spain [16]	Cross-sectional	Liver biopsy	ELISA	5	
Giannouli, 2023, Greece [17]	Case–control	Abdominal ultrasonography	ELISA	7	Adolescent population; all patients and controls with polycystic ovary syndrome
Goralska, 2023, Poland [18]	Cross-sectional	Non-invasive index of steatosis: FLI	ELISA	5	
Hua, 2019, Taiwan [19]	Cross-sectional	Abdominal ultrasonography	ELISA	7	Pediatric population
Ji, 2019, China [20]	Cross-sectional	Abdominal ultrasonography	ELISA	9	
Jiang, 2014, China [21]	Cross-sectional	Abdominal ultrasonography	ELISA	6	Patients and controls without T2DM
Ko, 2023, South Korea [22]	Cross-sectional	MRI-PDFF	ELISA	7	Pediatric population
Koliaki, 2015, Germany [23]	Cross-sectional	Liver biopsy	NA	6	Patients and controls with morbid obesity subjected to bariatric surgery
Koot, 2013, Netherlands [24]	Cross-sectional	MRS	ELISA	6	Pediatric and adolescent population
Li H, 2013, China [26]	Cross-sectional	Abdominal ultrasonography	ELISA	6	
Li X, 2011, China [27]	Cross-sectional	Abdominal ultrasonography	ELISA	7	Patients and controls without T2DM
Li X, 2024, China [28]	Cross-sectional	MAFLD criteria	ELISA	8	Patients and controls without T2DM
Lin D, 2023, China [29]	Cross sectional	Liver biopsy	ELISA	5	
Lin H, 2022, USA [30]	Cohort study	MRI	ELISA	9	Adolescent population; all patients and controls without T2DM
Liu, 2020, China [31]	Cross-sectional	Abdominal ultrasonography	ELISA	6	
Małecki, 2017, Poland [32]	Cross-sectional	Abdominal ultrasonography	ELISA	5	Pediatric population
Monserrat-Mesquida, 2020, Spain [33]	Cross-sectional	MRI-PDFF	ELISA	8	
Pafili, 2022, Germany [34]	Cross-sectional	Liver biopsy	ELISA	9	Patients and controls with morbid obesity subjected to bariatric surgery
Praktiknjo, 2019, Germany [35]	Cross-sectional	Transient elastography	ELISA	7	All patients and controls with HIV infection, without obesity
Qian, 2019, China [37]	Cross-sectional	MRS	ELISA	7	
Shen J, 2012, China [38]	Case–control	Liver biopsy	ELISA	8	
Shen Y, 2023, China [39]	Cohort study	Abdominal ultrasonography	ELISA	7	
Shen Y, 2013, China [40]	Cross-sectional	Abdominal ultrasonography	ELISA	6	
Singh, 2024, USA [41]	Cross-sectional	Liver Biopsy	ELISA	5	Patients, but not controls, with morbid obesity subjected to bariatric surgery
Sydor, 2022, Germany [42]	Cross-sectional	Transient elastography	ELISA	6	
Tanaka, 2022, Japan [43]	Cross-sectional	MAFLD criteria	ELISA	7	
Tucker, 2020, USA [44]	Cohort study	CT	ELISA	7	
Van Hove, 2024, USA [45]	Cross-sectional	Liver biopsy	ELISA	5	Pediatric patients but not controls diagnosed with NASH
Waluga, 2017, Poland [46]	Cross-sectional	Liver biopsy	ELISA	7	Patients and controls with morbid obesity subjected to bariatric surgery
Wargny, 2018, France [48]	Cohort study	MRI	ELISA	6	
Xu, 2024, China [49]	Cross-sectional	Abdominal ultrasonography and non-invasive indices	ELISA	5	
Yang, 2015, China [50]	Cross sectional	Liver biopsy	ELISA	8	
Yilmaz, 2010, Turkey [51]	Case–control	Liver biopsy	ELISA	7	

^†^: Studies are sorted alphabetically according to the surname of the first author. *CT* computed tomography, *ELISA* enzyme-linked immunosorbent assay, *FGF-21* fibroblast growth factor-21, *FLI* fatty liver index, *HIV* human immunodeficiency virus, *MAFLD* metabolic dysfunction-associated fatty liver disease, *MRI* magnetic resonance imaging, *MRI-PDFF* magnetic resonance imaging-proton density fat fraction, *MRS* magnetic resonance spectroscopy, *NA* not available, *NAFLD* nonalcoholic fatty liver disease, *NASH* nonalcoholic steatohepatitis, *NOS* Newcastle–Ottawa Scale, *PEI* Proximity extension immunoassay, *T2DM* type 2 diabetes mellitus

Demographic and laboratory characteristics extracted from the included studies are presented in the Table S1. For each study, data for the NAFLD and control groups were separately presented, including the number of patients (and men), age, BMI (kg/m^2^), waist circumference (cm), the number of patients with T2DM, FGF-21 levels (ng/ml), AST (IU/L), ALT (IU/L), GGT (IU/L), HOMA-IR, and the number of patients with NASH-related cirrhosis.

### Quality of the Included Studies

The NOS score was used for the evaluation of all studies (Table 1). There were 19 studies (43.2%) with NOS score < 7. The mean (± SD) NOS score was 6.68 (± 1.30).

### Main and Secondary Outcomes

**Fig. 3:**
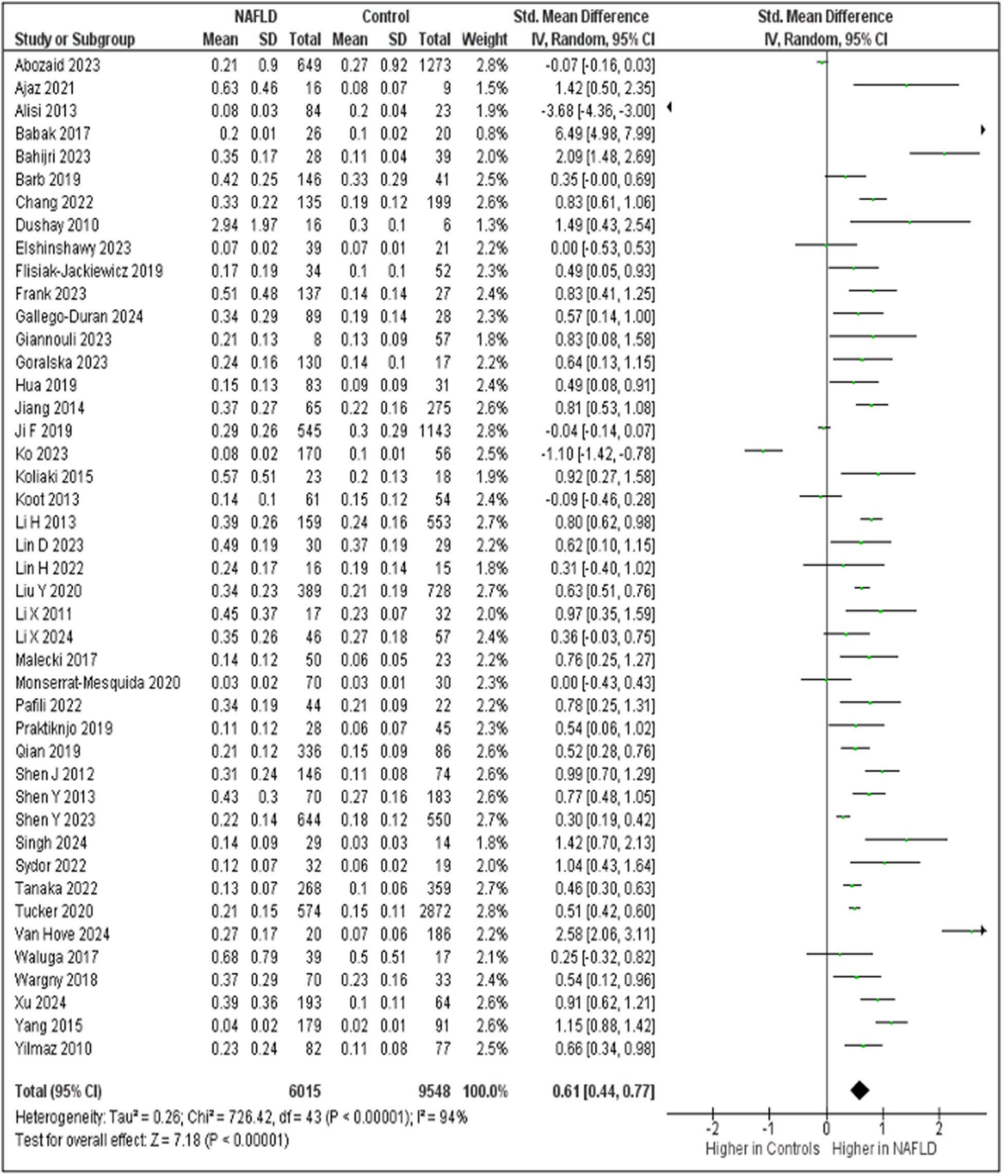
Forest plot for the comparison of circulating FGF-21 between patients with NAFLD and controls in all studies (n = 44) included in the meta-analysis. Abbreviations: CI, confidence intervals; FGF-21, fibroblast growth factor-21; IV, inverse variance; NAFLD, nonalcoholic fatty liver disease; SD, standard deviation

Circulating FGF-21 was higher in patients with NAFLD compared to controls (SMD: 0.61; 95% CI: 0.44, 0.77; P < 0.00001) (Table 2; Fig. 3). Heterogeneity among studies was high (I^2^ = 94%). Egger’s test suggested statistically significant publication bias (p = 0.030) and visual asymmetry was observed in the relevant funnel plot (Fig. S1). Subgroup comparisons according to the disease severity in studies with histological confirmation showed: (i) no statistical difference in circulating FGF-21 between patients with NAFL and controls (n = 9; SMD: 0.22; 95% CI: −0.49, 0.93; p = 0.540; Table 3; Fig. S2a-b); (iii) higher FGF-21 levels in patients with NASH compared to controls (n = 12; SMD: 1.30; 95% CI: 0.35, 2.24; P = 0.007; Table 3; Fig. S2c-d); no statistical difference in circulating FGF-21 between patients with NAFL and patients with NASH (n = 9; SMD: 1.17; 95% CI: −0.06, 2.39; p = 0.060; Table 3; Fig. S2e-f). Heterogeneity in these three subgroup comparisons remained high, whereas Egger’s test showed not significant publication bias, which was confirmed by the visualization of funnel plots.

**Table 2:** FGF-21 SMD and related statistics between patients with NAFLD and controls in the sum of studies and in sensitivity analyses

Comparison NAFLD versus Controls	All studies (n = 44)	After excluding studies with pediatric/adolescent populations (n = 35)	After excluding studies with populations undergoing bariatric surgery (n = 40)	After excluding studies with NOS < 7 (n = 25)	After excluding studies with outliers of FGF-21 SMD (N = 41)	After excluding studies with the use of the definition of MAFLD (n = 42)
SMD (95% CI); p-value	0.61 (0.44, 0.77); < 0.00001	0.70 (0.55, 0.85); < 0.00001	0.59 (0.41, 0.76); < 0.00001	0.37 (0.17, 0.58); 0.0004	0.58 (0.44, 0.72); < 0.00001	0.62 (0.44, 0.79); < 0.00001
I^2^; p-value	94%; < 0.00001	92%; < 0.00001	95%; < 0.00001	95%; < 0.00001	91%; < 0.00001	94%; < 0.00001
Egger’s test p-value	0.030	0.0004	0.049	0.422	0.012	0.031

*CI* confidence interval, *FGF-21* fibroblast growth factor-21, *MAFLD* metabolic dysfunction-associated fatty liver disease, *NAFLD* nonalcoholic fatty liver disease, *NOS* Newcastle–Ottawa Scale, *n* number of studies included in the analysis, *SMD* standardized mean difference

**Table 3:** FGF-21 SMD and related statistics in subgroup analysis according to NAFLD severity

	NAFL vs. Controls (n = 9)	NASH vs. Controls (n = 12)	NASH vs. NAFL (n = 9)
SMD (95% CI); p-value	0.22 (−0.49, 0.93); p = 0.540	1.30 (0.35, 2.24); p = 0.007	1.17 (-.0.06, 2.39); p = 0.060
I^2^; p-value	95%; p < 0.00001	97%; p < 0.00001	98% p < 0.00001
Egger’s test p-value	0.795	0.999	0.579

*CI* confidence interval, *FGF-21* fibroblast growth factor-21, *NAFLD* nonalcoholic fatty liver disease, *n* number of studies, *SMD* standardized mean difference

In the subgroup analysis for the comparison between patients with NAFLD and controls among studies with and without histological confirmation of the disease, there was no difference between subgroups (p = 0.590); circulating FGF-21 was higher in patients with NAFLD than controls within both subgroups (Table 4; Fig. S2 g-i). Heterogeneity remained high in both subgroup comparisons. In the subgroup analysis for the comparison between patients with NAFLD and controls within studies with and without the inclusion of patients with NASH-related liver cirrhosis, there was statistically significant difference between subgroups (p = 0.0004), with FGF-21 SMD being higher in studies with the inclusion of patients with NASH-related cirrhosis (SMD: 1.01, 95% CI: 0.84, 1.18, p < 0.00001; Table 4; Fig. S2j-l). Notably, heterogeneity radically decreased in the subgroup of studies including patients with NASH-related cirrhosis (I^2^ = 0%; p = 0.480), whereas it remained high in the absence of patients with NASH-related cirrhosis (Table 4; Fig. S2j). No significant publication bias was suggested by Egger’s test and funnel plots (Table 4; Fig. S2 h-i, S2 k-l).

**Table 4:** FGF-21 SMD and related statistics between patients with NAFLD and controls in subgroup analysis within studies: (i) with vs. without histological confirmation of NAFLD with liver biopsy and (ii) with vs. without the inclusion of patients with NASH-related cirrhosis

	Liver Biopsy (n = 15)	Without Liver Biopsy (n = 29)	NASH related cirrhosis (n = 4)	Without NASH related cirrhosis (n = 40)
SMD (95% CI); p-value	0.69 (0.20, 1.17); 0.006	0.54 (0.37, 0.72); < 0.00001	1.01 (0.84, 1.18); < 0.00001	0.57 (0.40,0.74); < 0.00001
I^2^; p-value	94%; < 0.00001	94%; < 0.00001	0%; 0.48	94%; < 0.00001
Egger’s test p-value	0.599	0.053	0.103	0.062
p-value for difference (between subgroups)	0.590		0.0004	

*CI* confidence interval, *FGF-21* fibroblast growth factor-21, *NAFLD* nonalcoholic fatty liver disease, *n* number of studies, *SMD* standardized mean difference

In the sensitivity analyses for the comparison between patients with NAFLD and controls, after excluding studies with: (i) pediatric/adolescent populations (n = 9; Fig. S3a); (ii) morbidly obese populations undergoing bariatric surgery (n = 4; Fig. S3b); (iii) NOS score < 7 (n = 19; Fig. S3c); (iv) outliers of FGF-21 SMD (n = 3; Fig. S3 d); (v) the use of the definition of MAFLD for the diagnosis of the disease (n = 2; Fig. S3e), FGF-21 levels remained higher in patients with NAFLD compared to controls in all the analyses (Table 2). Heterogeneity remained essentially unchanged in all the sensitivity analyses. Egger’s test p-value remained statistically significant in all comparisons, except for that following the exclusion of studies with NOS score < 7 (p = 0.422). Visual inspection of funnel plots indicated a degree of asymmetry (Fig. S4a-e).

In the univariate meta-regression analysis, the percentage of patients with T2DM was positively associated with FGF-21 SMD between patients with NAFLD and controls and could explain 49.2% of the heterogeneity among studies (Table 5; Fig. S5). The rest of the selected potential confounders, i.e., age, sex, BMI, waist circumference and HOMA-IR were not significantly associated with FGF-21 SMD between patients with NAFLD and controls (Table 5). Thus, these parameters could not explain a part of the heterogeneity among studies.

**Table 5:** Univariate meta-regression analysis of FGF-21 SMD between NAFLD patients and controls with potential confounders

Confounder	Age (years)	Sex (men%)	T2DM (%)	BMI (kg/m^2^)	Waist Circumference (cm)	HOMA-IR
N	32	31	14	32	16	14
Beta (95%CI)	0.013 (−0.005, 0.031)	−1.414 (−3.685, 0.856)	1.043 (0.400, 1.686)	0.022 (−0.023, 0.066)	0.012 (−0.012, 0.036)	0.038 (−0.125, 0.202)
p-value	0.158	0.222	0.0015	0.339	0.328	0.647
Adjusted R square	2.74%	1.52%	49.18%	0.00%	0.00%	0.00%

*BMI* body mass index, *CI* confidence interval, *HOMA-IR* homeostasis model assessment-insulin resistance, *FGF-21* fibroblast growth factor-21, *NAFLD* nonalcoholic fatty liver disease, *N* number of studies with available data on each potential confounder, *SMD* standardized mean difference, *T2DM* type 2 diabetes mellitus

## Discussion

This systematic review and meta-analysis demonstrated higher circulating FGF-21 in patients with NAFLD compared to controls (Table 2; Fig. 3). Within the subgroup of studies with histological confirmation of the disease, FGF-21 levels were higher in patients with NASH than controls, but not in patients with NAFL compared to controls or patients with NASH [Table 3; Fig. S2a, S2c, S2e]. Notably, in another subgroup analysis, higher FGF-21 between patients with NAFLD and controls was observed within studies that included patients with NASH-related cirrhosis than those that did not include them (Table 4; Fig. S2j). Altogether these findings indicate that higher circulating FGF-21 levels observed in patients with NAFLD than controls may be primarily attributed to more severe disease; however, the interpretation of this result should be approached with caution, because of the relatively small number of studies with histological confirmation or with the inclusion of patients with NASH-related cirrhosis.

Trying to investigate the high heterogeneity among studies, subgroup, sensitivity and meta-regression analyses were performed. The inclusion of patients with NASH-related cirrhosis, i.e., the whole histological spectrum of NAFLD minimized the heterogeneity (Table 4; Fig. S2j). This implies that not inclusion of the full spectrum of NALFD in the relevant studies is partly accounted for the observed heterogeneity; however, the interpretation of this result should also be approached with caution, due to the small number of studies having included patients with NASH-related cirrhosis. Beyond this finding, the other subgroup and sensitivity analyses did not essentially reduce the heterogeneity among studies (Tables 2–4). The meta-regression analysis showed that the percentage of patients with T2DM may be accounted for about 49% of the heterogeneity among studies (Table 5; Fig. S5). This seems rational since higher rates of T2DM were observed in individuals with than without NAFLD [57], and FGF-21 was shown higher in patients with than without T2DM [58]. Nonetheless, this finding should be cautiously interpreted, because of the small number of studies with data on T2DM in this meta-analysis (n = 14). The other potential confounders, i.e., age, sex, BMI, waist circumference and HOMA-IR could not explain a part of heterogeneity among studies. Based on the above findings, the inclusion of patients across the full spectrum of NAFLD and the number of patients with T2DM are highly recommended in the future relevant studies.

Egger’s test showed an overall publication bias in the comparison between patients with NAFLD and controls and most funnel plots also showed a degree of asymmetry (Fig. S2b, S2 d, S2f, S2 h-i, S2k-l, S4a-e). However, Egger’s test was not significant and the funnel plot showed lower degree of asymmetry in all subgroup analyses (Tables 3–4), as well as in the sensitivity analysis after the exclusion of studies with NOS < 7, i.e., those with estimated lower quality. The latter finding may imply that most relevant studies of higher quality, i.e., those providing the most reliable findings, were included in this systematic review and meta-analysis.

From a pathophysiologic point of view, the production of FGF-21 may increase when NAFLD progresses to more advanced disease, as a counterbalancing mechanism against the disease progression [8]. Data from experimental studies have shown that FGF-21, acting through on its receptor (FGFR1) on the cell membrane with β-Klotho as co-receptor, increases fatty acid β-oxidation and decreases de novo lipogenesis, thereby attenuating hepatic steatosis. Additionally, FGF-21 may attenuate hepatic inflammation by inhibiting the nuclear factor kappa B pathway, decreasing pro-inflammatory cytokines (e.g., interleukin-1β) and increasing anti-inflammatory cytokines (e.g., interleukin-10), decreasing intrahepatic oxidative stress and endoplasmic reticulum stress. FGF-21 may also possibly decrease hepatic fibrosis, by attenuating the hepatic expression of transforming growth factor-β [8]. Notably, FGF-21 seems to increase insulin sensitivity in the liver [59].

However, higher FGF-21 concentrations in advanced disease may also imply a state of FGF-21 resistance or insensitivity [8]. In this case, the administration of FGF-21 analogs, in order to achieve supraphysiological concentrations of FGF-21, thus surpassing the barrier of FGF-21 resistance or insensitivity, may be beneficial, thus limiting the progression of the disease, or may fail to limit the progression of the disease. Indeed, FGF-21 analogs (e.g., pegbelfermin, efruxifermin) have been investigated in clinical trials of NASH. In two phase 2b RCTs, pegbelfermin was administered in patients with NASH and advanced hepatic fibrosis (FALCON 1 trial) [60] or NASH-associated compensated cirrhosis (FALCON 2 trial) [61], without, however meeting its primary endpoints. On the contrary, in a phase 2b RCT, efruxifermin administration in patients with NASH and moderate or advanced hepatic fibrosis (HARMONY) provided more favorable results; more specifically, efruxifermin improved hepatic fibrosis and resolved NASH in higher rates than placebo [62]. The above considering, the main clinical implication of our meta-analysis is the consolidation of higher circulating FGF-21 concentrations, when the disease progresses to NASH or NASH-related cirrhosis. Whether the administration of FGF-21 analogs to achieve supraphysiological FGF-21 concentrations are definitely beneficial or not for NASH may hopefully be shown by the ongoing clinical trials.

There are some meta-analyses of clinical trials investigating the effects of FGF-21 analogs on NAFLD. In a meta-analysis of 8 RCTs, FGF-21 analogs were shown to reduce NAFLD activity score (NAS) (without worsening of fibrosis) and fibrosis stage (without worsening of MASH) [63]. Similar results were provided by other relevant meta-analysis of 7 [64] or 6 [65] clinical trials. The results of the existing meta-analyses should cautiously be interpreted, because of the small number of included studies, which, importantly, were mostly sponsored. Furthermore, different FGF-21 analogs (e.g., efruxifermin, pegbelfermin, and pegozafermin) were synthesized together, which renders difficult the interpretation of the results of the relevant meta-analyses. There is also a meta-analysis of diagnostic accuracy, which supported that FGF-21 provided a pooled sensitivity of 0.62 (95% CI 0.50–0.73) and specificity of 0.78 (0.70–0.84) for diagnosing NASH, based on the results of four studies [66]. However, we could not recommend the use of FGF-21 as a non-invasive index of NASH, based on the results of this [66] or our meta-analysis; of course, our results warrant more diagnostic accuracy studies to clarify whether FGF-21 may add value in the non-invasive diagnosis of NASH or related fibrosis, alone or, more possibly, in combination with other parameters.

There is also a recent bi-directional Mendelian randomization study supporting that FGF-21 was negatively regulated by NAFLD, whereas positively regulated by obesity and T2DM [67]. However, FGF-21 did not improve NAFLD, obesity or T2DM, possibly owing to FGF-21 resistance [67]. This study contradicts the main result of our meta-analysis, i.e., higher FGF-21 in patients with NAFLD than controls; however, the reasons why FGF-21 was differently regulated by NAFLD and obesity or T2DM were not clear, since their possible effects on FGF-21 are expected to be towards the same direction. The authors of this study also supported that FGF-21 resistance may inhibit any counterbalancing effects of FGF-21 changes not only in NAFLD, but also in obesity and T2DM, which is in accordance with our speculation above.

This systematic review and meta-analysis investigating circulating FGF-21 levels in patients with NAFLD carries a degree of originality. Furthermore, our findings are highly relevant with the above-mentioned clinical trials investigating FGF-21 analogs for the treatment of NASH, as mentioned above, especially in the light of potential FGF-21 resistance or insensitivity observed in advanced disease. However, this systematic review and meta-analysis has certain limitations. First, the inclusion of observational studies cannot show a cause-effect association between FGF-21 and NAFLD. Second, the comparison of circulating FGF-21 levels between different grades of hepatic steatosis, different stages of hepatic fibrosis and different degree of hepatic inflammation was not feasible, because of the very small number of studies providing the specific histological information; even when FGF-21 levels were reported, the groups were differently defined in different studies. For example, when reported, fibrosis stages were grouped as F0 vs. F1-F4, or F0-1 vs. F2-F3, or F0-2 vs. F3-4 etc. in different studies, which rendered the synthesis of grouping for fibrosis stages insecure; however, FGF-21 levels were higher in NAFLD patients than controls among studies having included patients with NASH-related cirrhosis, which equals with fibrosis stage F4 (Table 4; Fig. S2j), thus providing an indirect indication of higher FGF-21 levels in the end stage of fibrosis. Third, the pre-planned subgroup analysis based on different methods of FGF-21 measurement was not feasible, because all but two studies utilized ELISA for FGF-21 quantification; however, different ELISA kits from different manufacturers might have affected the heterogeneity among studies. Fourth, heterogeneity among studies was high and could be explained only partly by the sensitivity analyses (Table 4; Fig. S2j) and meta-regression analyses (Table 5; Fig. S5). Furthermore, Egger’s test showed an overall publication bias, despite the extensive manual searching we performed. Last, we adopted the nomenclature of NAFLD and not that of MASLD that has been more recently suggested, because, as mentioned above, most studies included in the systematic review and meta-analysis were based on the nomenclature and definition of NAFLD (Table 1). Although there is significant overlap between NAFLD, MAFLD and MASLD, the proposed shift from NAFLD to MAFLD or MASLD should cautiously be performed because of the different definitions among them [68]. In this regard, it has been recommended that all different names of the disease may be used during this period of transition with the necessary flexibility [69].

## Conclusions

Higher circulating FGF-21 levels were shown in patients with NAFLD, which may be possibly attributed to those with advanced disease (NASH and or NASH-related cirrhosis). These results may imply a counterbalancing increase of FGF-21, when NAFLD progresses to advanced disease, thus possibly supporting the ongoing clinical trials of FGF-21 analogs in patients with NASH. However, FGF-21 resistance or insensitivity, when the disease progresses may also be considered, a condition which, if validated, may interfere with the results of the relevant clinical trials.

## Key References

• Fouad Y, Alboraie M, Shiha G. Epidemiology and diagnosis of metabolic dysfunction-associated fatty liver disease. Hepatol Int. 2024;18:827–33. 10.1007/s12072-024-10704-3.
  This review provides a contemporary summary of the epidemiology and the diagnosis of the disease.
•• Eslam M, Newsome PN, Sarin SK, Anstee QM, Targher G, Romero-gomez M, et al. A new definition for metabolic dysfunction-associated fatty liver disease : An international expert consensus statement. J Hepatol. 2020;73:202–9. 10.1016/j.jhep.2020.03.039.
  This consensus introduced the nomenclature and the definition of metabolic dysfynction-associated fatty liver disease.
•• Rinella ME, Lazarus JV, Ratziu V, Francque SM, Sanyal AJ, Kanwal F, et al. A multisociety Delphi consensus statement on new fatty liver disease nomenclature. J Hepatol. 2023;79:1542–56. 10.1016/j.jhep.2023.06.003.
  This Delphi consensus introduced the nomenclature and the definition of metabolic dysfunction‑associated steatotic liver disease.
• Loomba R, Sanyal AJ, Nakajima A, Neuschwander-Tetri BA, Goodman ZD, Harrison SA, et al. Pegbelfermin in Patients With Nonalcoholic Steatohepatitis and Stage 3 Fibrosis (FALCON 1): A Randomized Phase 2b Study. Clin Gastroenterol Hepatol. 2024;22:102–12. 10.1016/j.cgh.2023.04.011.
  This is a phase 2b clinical trial investigating the effect of pegbelfermin, a FGF-21 analog in patient with steatohepatitis and advanced fibrosis.
• Abdelmalek MF, Sanyal AJ, Nakajima A, Neuschwander-Tetri BA, Goodman ZD, Lawitz EJ, et al. Pegbelfermin in Patients With Nonalcoholic Steatohepatitis and Compensated Cirrhosis (FALCON 2): A Randomized Phase 2b Study. Clin Gastroenterol Hepatol. 2024;22:113–23. 10.1016/j.cgh.2023.04.012.
  This is a phase 2b clinical trial investigating the effect of pegbelfermin, an FGF-21 analog in patient with steatohepatitis and compansated cirrhosis.
• Harrison SA, Frias JP, Neff G, Abrams GA, Lucas KJ, Sanchez W, et al. Safety and efficacy of once-weekly efruxifermin versus placebo in non-alcoholic steatohepatitis (HARMONY): a multicentre, randomised, double-blind, placebo-controlled, phase 2b trial. Lancet Gastroenterol Hepatol. 2023;8:1080–93. 10.1016/S2468-1253(23)00272-8.
  This is a phase 2b clinical trial investigating the effect of efruxifermin, a FGF‑21 analog in patient with steatohepatitis.

## Supplementary Information

Below is the link to the electronic supplementary material. 

ESM1 (PPTX 47.9 KB) 

ESM2 (PPTX 217 KB) 

ESM3 (PPTX 191 KB) 

ESM4 (PPTX 78.7 KB) 

ESM5 (PPTX 52.1 KB) 

ESM6 (DOCX 107 KB)

ESM7 (DOCX 28.2 KB)

## Data Availability

Data are provided within the manuscript and the supplementary information files. If needed, any additional data will be provided by the corresponding authors upon reasonable request.

## Abbreviations

AASLD: American Association for the Study of Liver Diseases
ALT: Alanine Aminotransferase
APASL: Asian-Pacific Association for the Study of the Liver
AST: Aspartate Aminotransferase
BMI: Body Mass Index
CI: Confidence Interval
CT: Computed Tomography
EASD: European Association for the Study of Diabetes
EASL: European Association for the Study of the Liver
EASO: European Association for the Study of Obesity
ELISA: Enzyme-Linked Immunosorbent Assay
FGF-21: Fibroblast growth factor-21
GGT: Gamma-Glutamyl Transferase
HOMA-IR: Homeostasis Model Assessment-Insulin Resistance
IR: Insulin Resistance
MAFLD: Metabolic (dysfunction-)Associated Fatty Liver Disease
MASLD: Metabolic dysfunction-Associated Steatotic Liver Disease
MASH: Metabolic dysfunction-Associated Steatohepatitis
MeSH: Medical Subject Headings
MOOSE: Meta-analysis of Observational Studies in Epidemiology
MRE: Magnetic Resonance Elastography
MRI: Magnetic Resonance Imaging
MRI-PDFF: Magnetic Resonance Imaging-proton density fat fraction
MRS: Magnetic Resonance Spectroscopy
NA: Not Available
NAFL: Nonalcoholic Fatty Liver
NAFLD: Nonalcoholic Fatty Liver Disease
NAS: NAFLD Activity Score
NASH: Nonalcoholic Steatohepatitis
NOS: Newcastle-Ottawa Scale
PECO: Population, Exposure, Comparison, Outcome
PEI: Proximity Extension Immunoassay
PRISMA: Preferred Reporting Items for Systematic Reviews and Meta-Analyses
RCT: Randomized Controlled Trial
SD: Standard Deviation
SMD: Standardized Mean Difference
T2DM: Type 2 Diabetes Mellitus
