# Supplementary figures and images for: Circulating Fibroblast Growth Factor-21 in Patients with Nonalcoholic Fatty Liver Disease: A Systematic Review and Meta-Analysis
Source: Curr Obes Rep. 2025 Jun 4;14(1):51. doi: 10.1007/s13679-025-00643-x (PMC12137391; doi:10.1007/s13679-025-00643-x)

## Slide 1
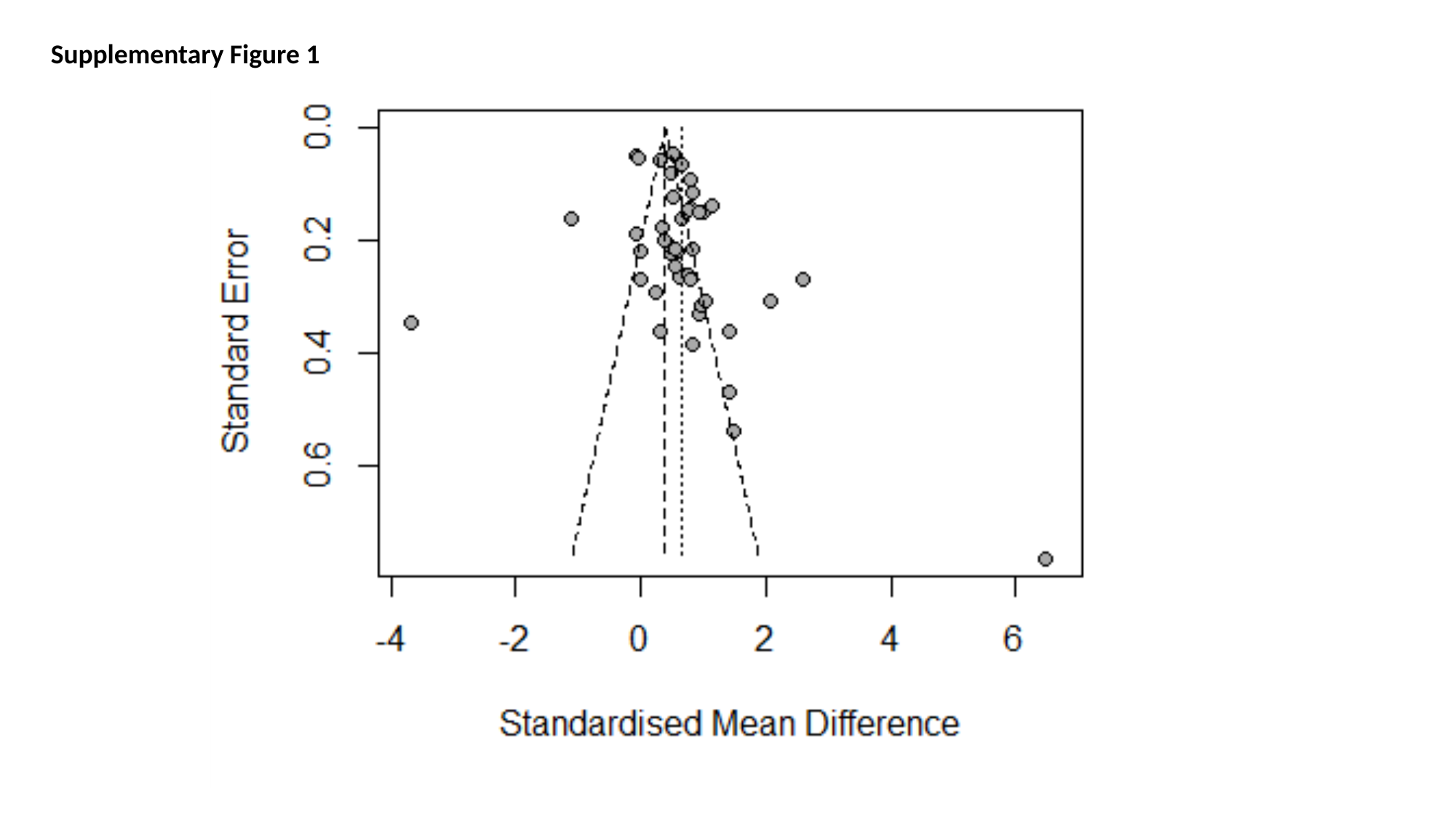

Supplementary Figure 1

Supplement: Supplementary file 1 — (PPTX 47.9 KB) [file 13679_2025_643_MOESM1_ESM.pptx]

## Slide 1
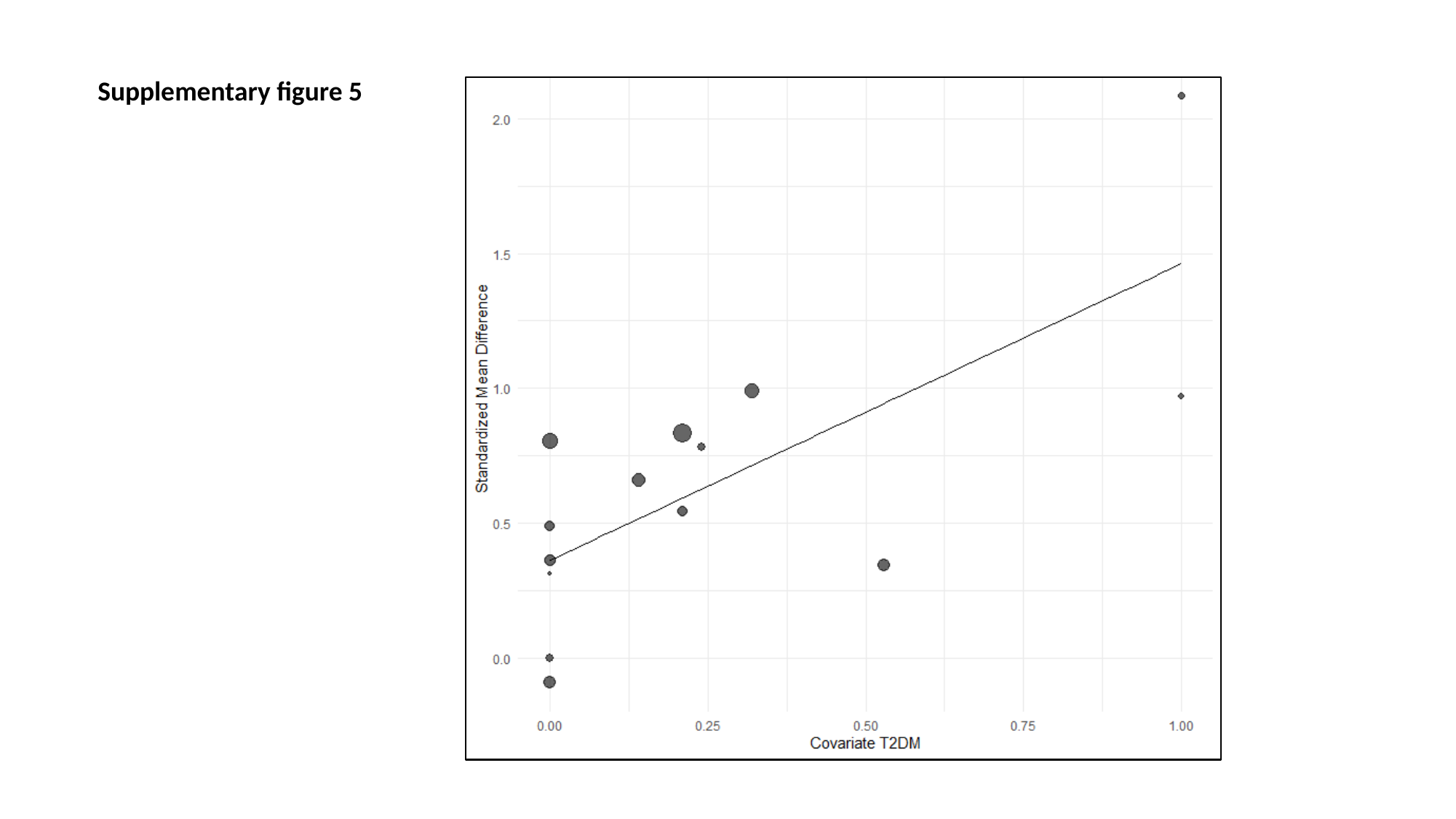

Supplementary figure 5

Supplement: Supplementary file 5 — (PPTX 52.1 KB) [file 13679_2025_643_MOESM5_ESM.pptx]
