# Supplementary material for: Circulating Fibroblast Growth Factor-21 in Patients with Nonalcoholic Fatty Liver Disease: A Systematic Review and Meta-Analysis
Source: Curr Obes Rep. 2025 Jun 4;14(1):51. doi: 10.1007/s13679-025-00643-x (PMC12137391; doi:10.1007/s13679-025-00643-x)
Supplement: Supplementary file 2 — (PPTX 217 KB) [file 13679_2025_643_MOESM2_ESM.pptx]

## Slide 1
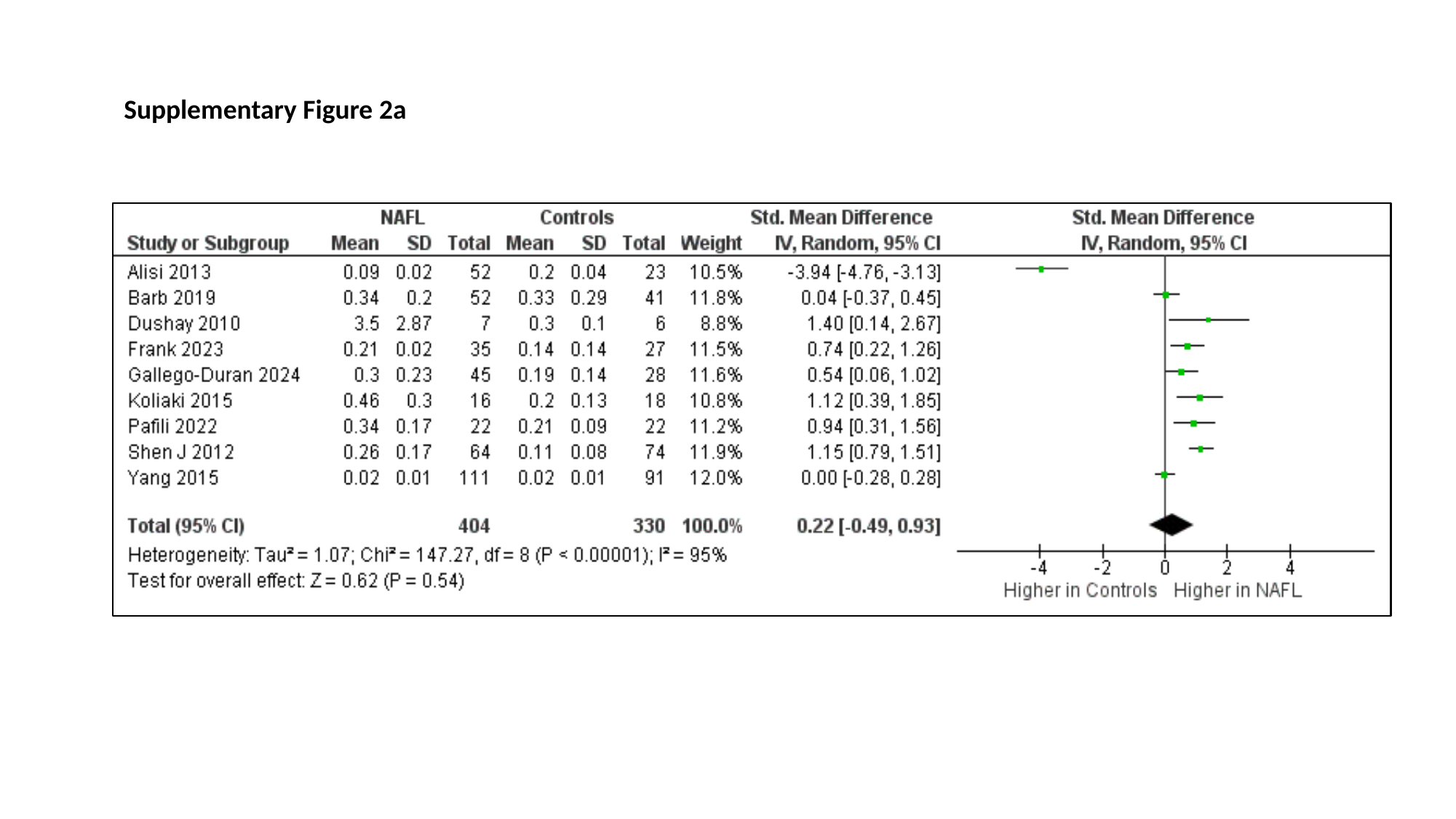

Supplementary Figure 2a

## Slide 2
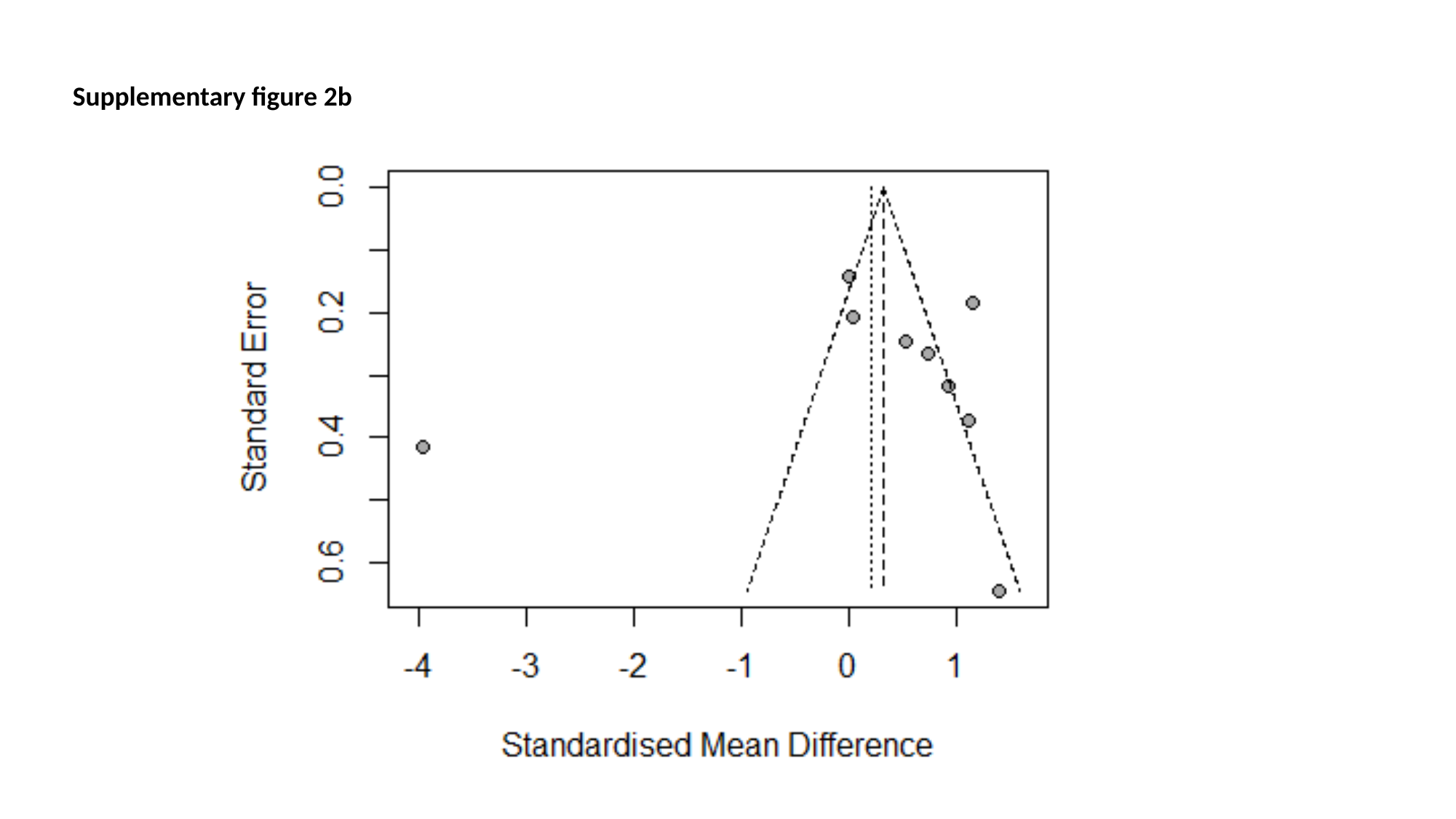

Supplementary figure 2b

## Slide 3
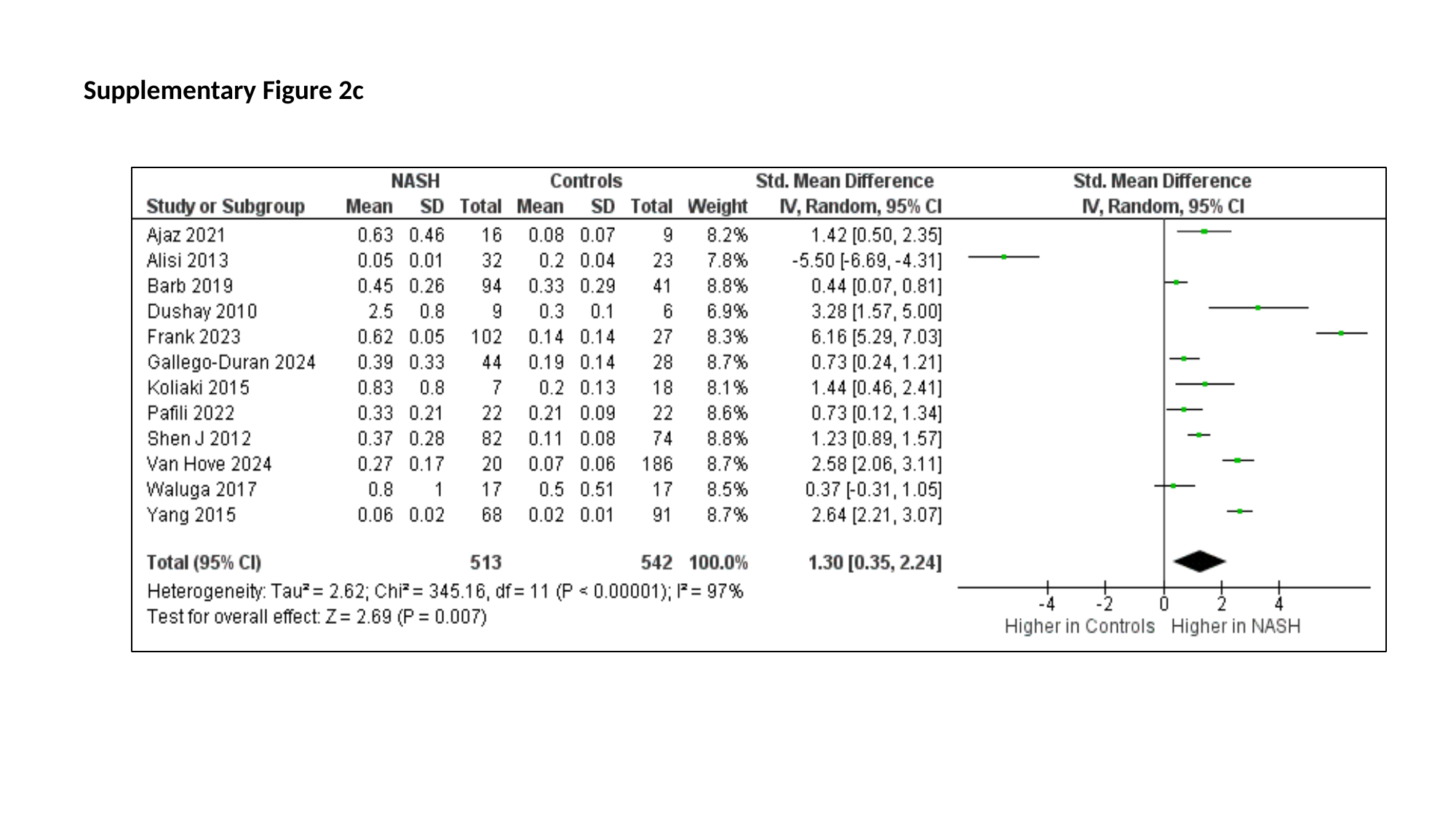

Supplementary Figure 2c

## Slide 4
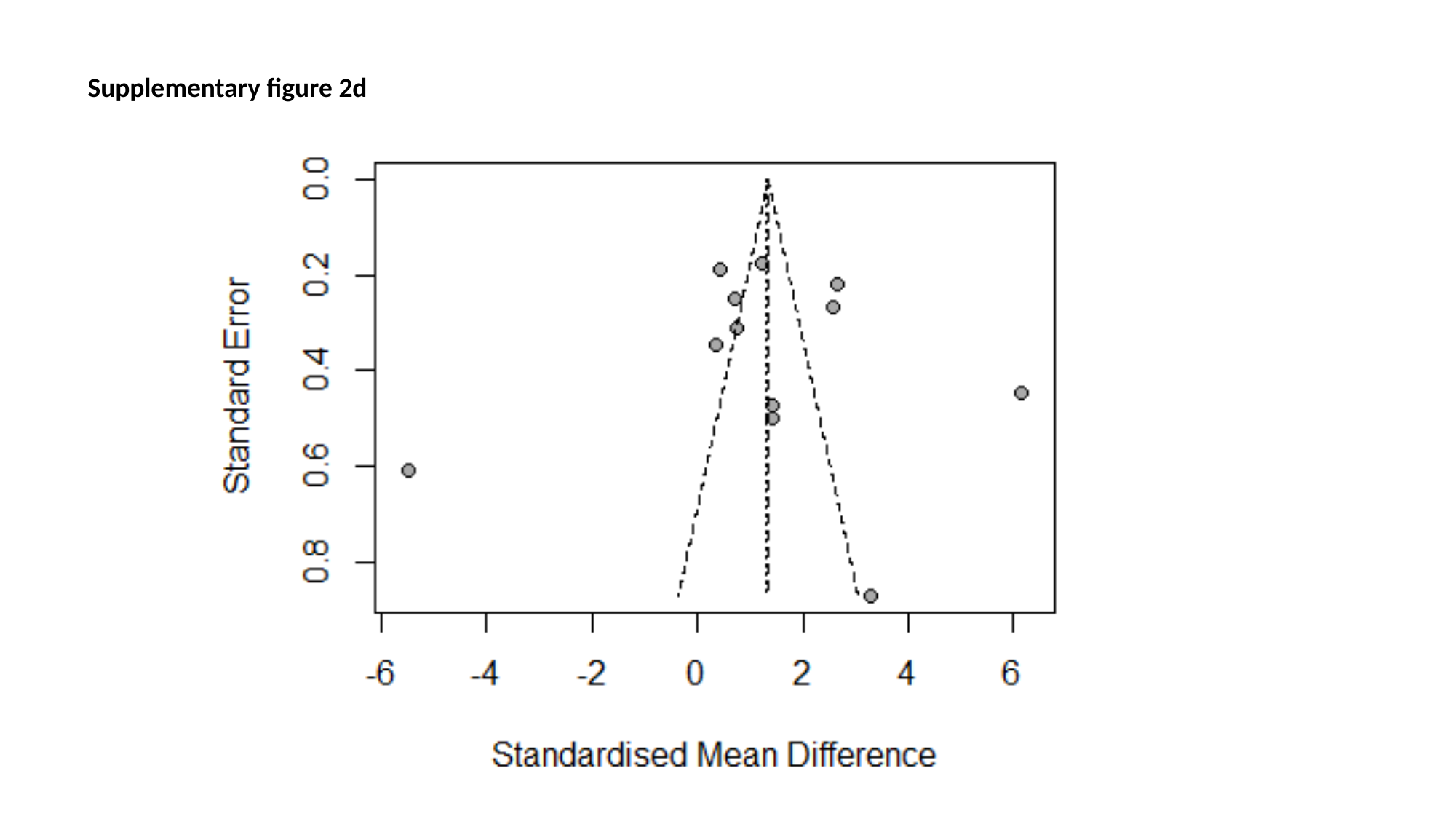

Supplementary figure 2d

## Slide 5
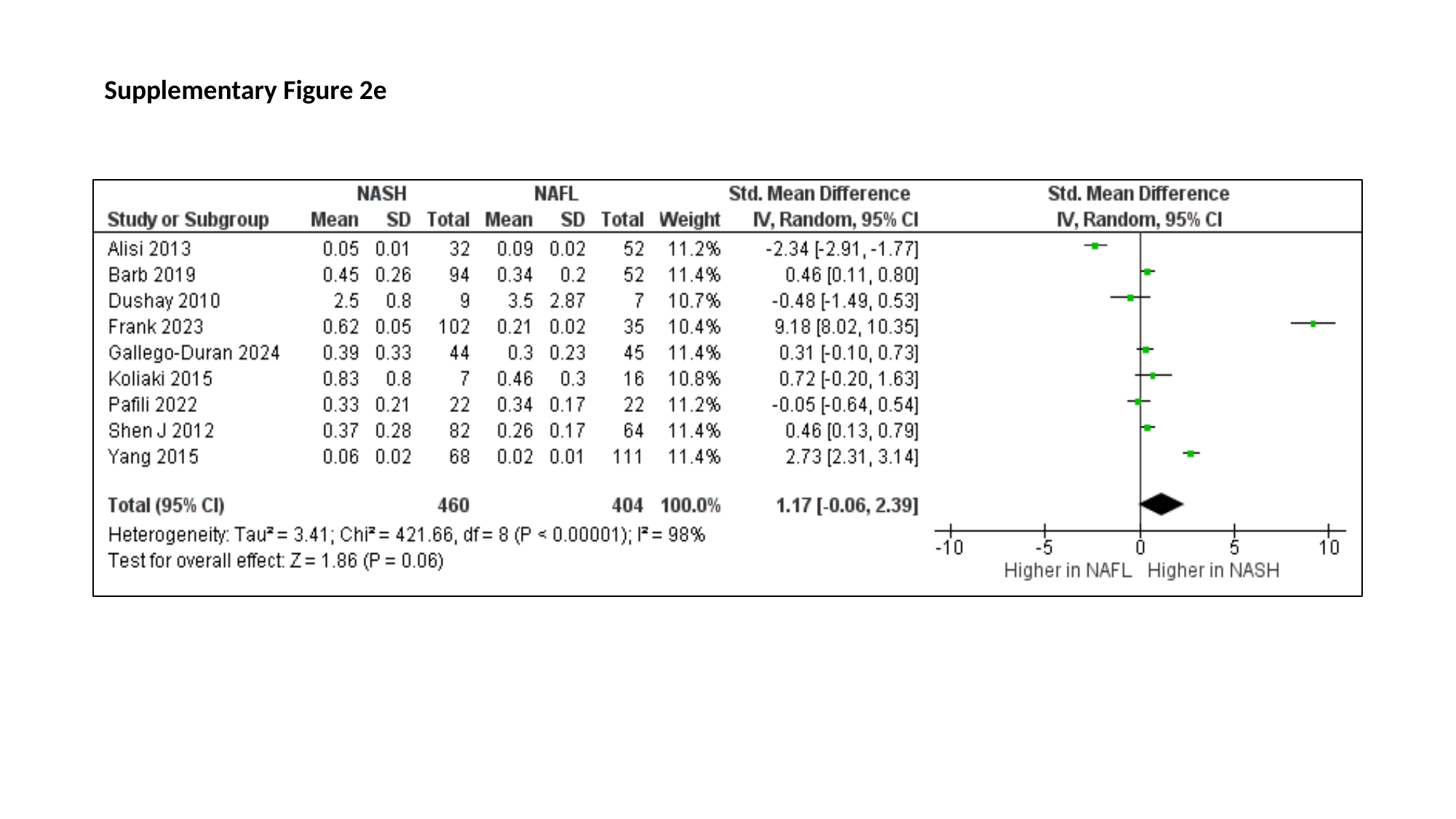

Supplementary Figure 2e

## Slide 6
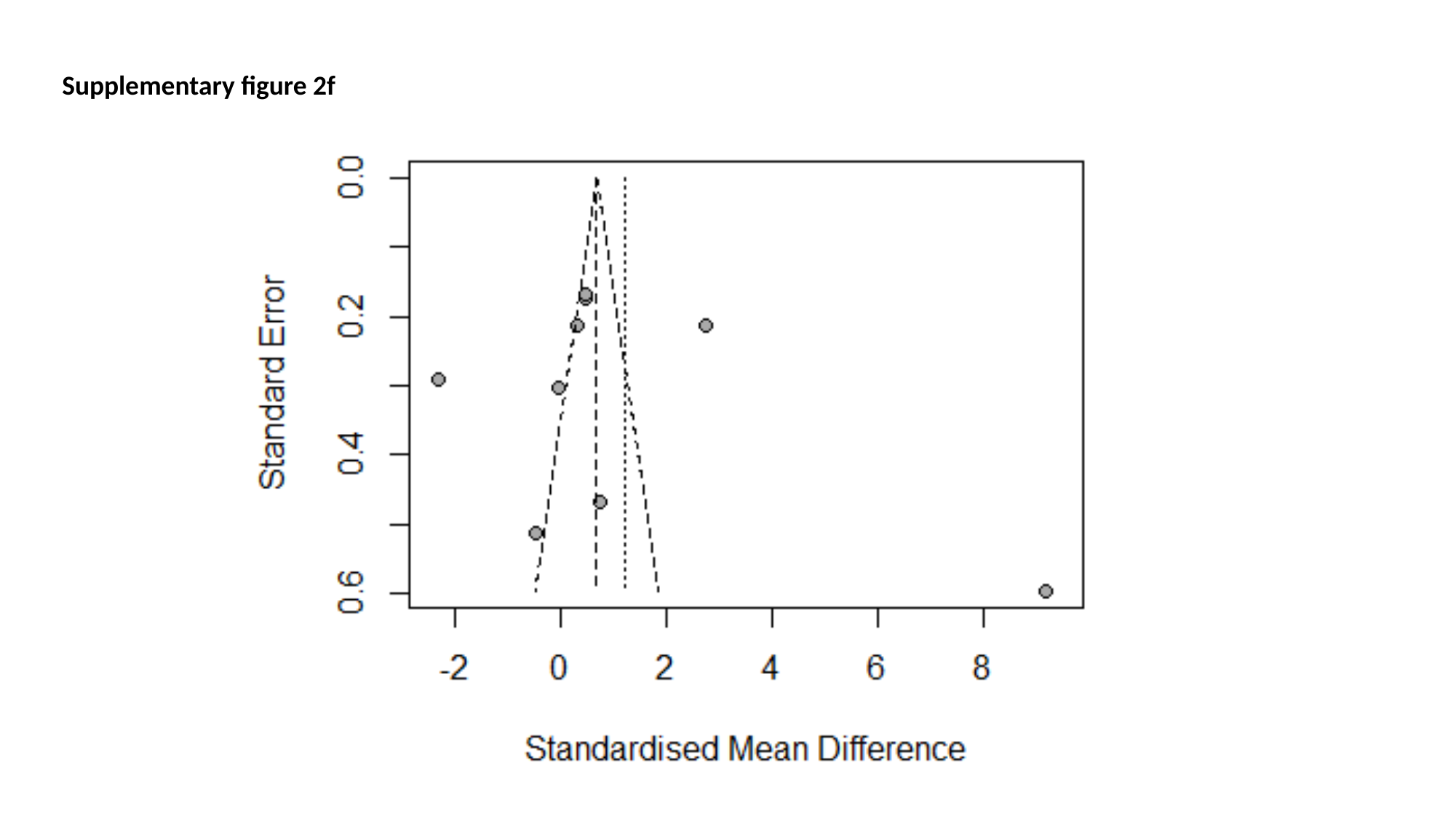

Supplementary figure 2f

## Slide 7
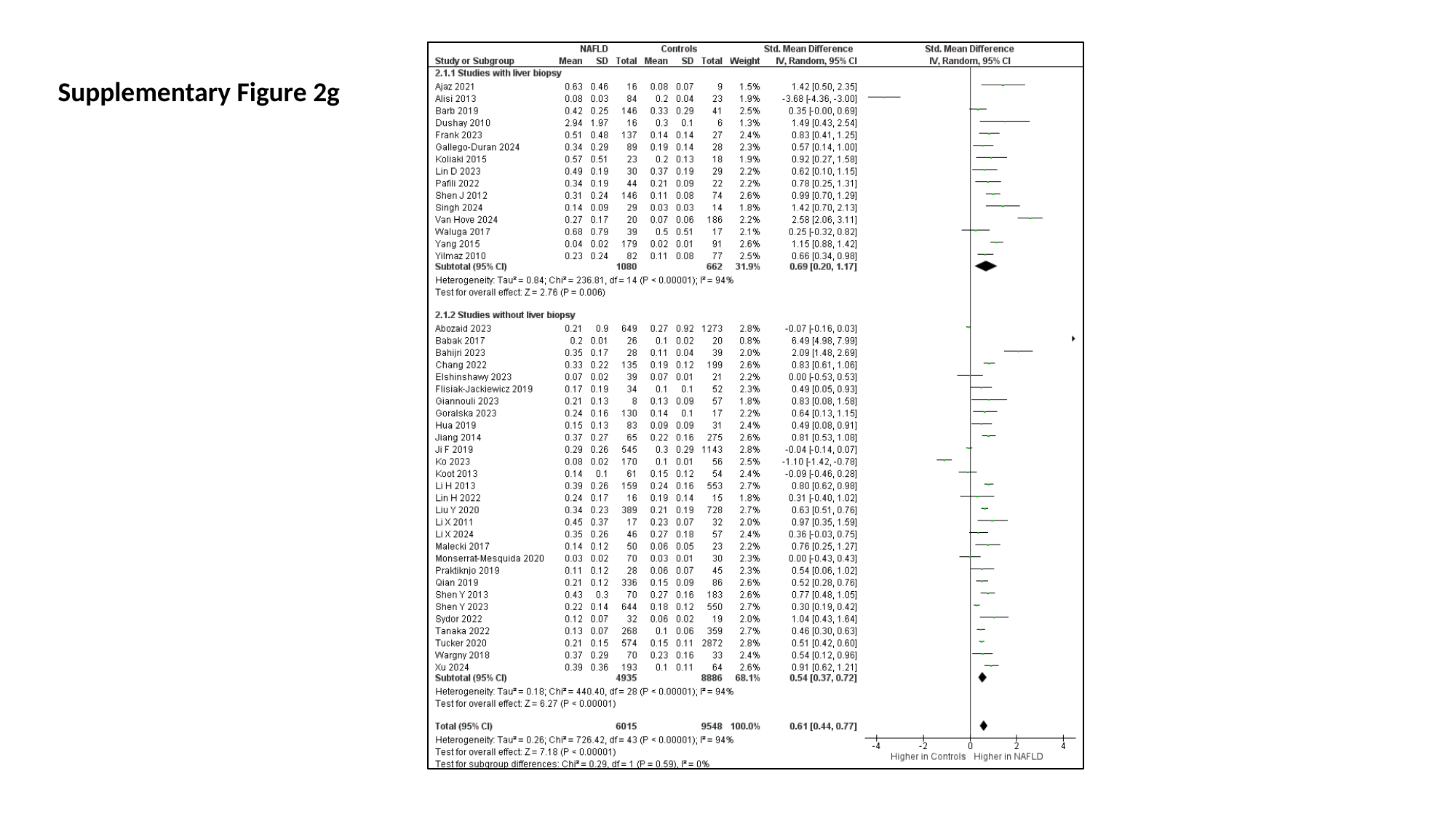

Supplementary Figure 2g

## Slide 8
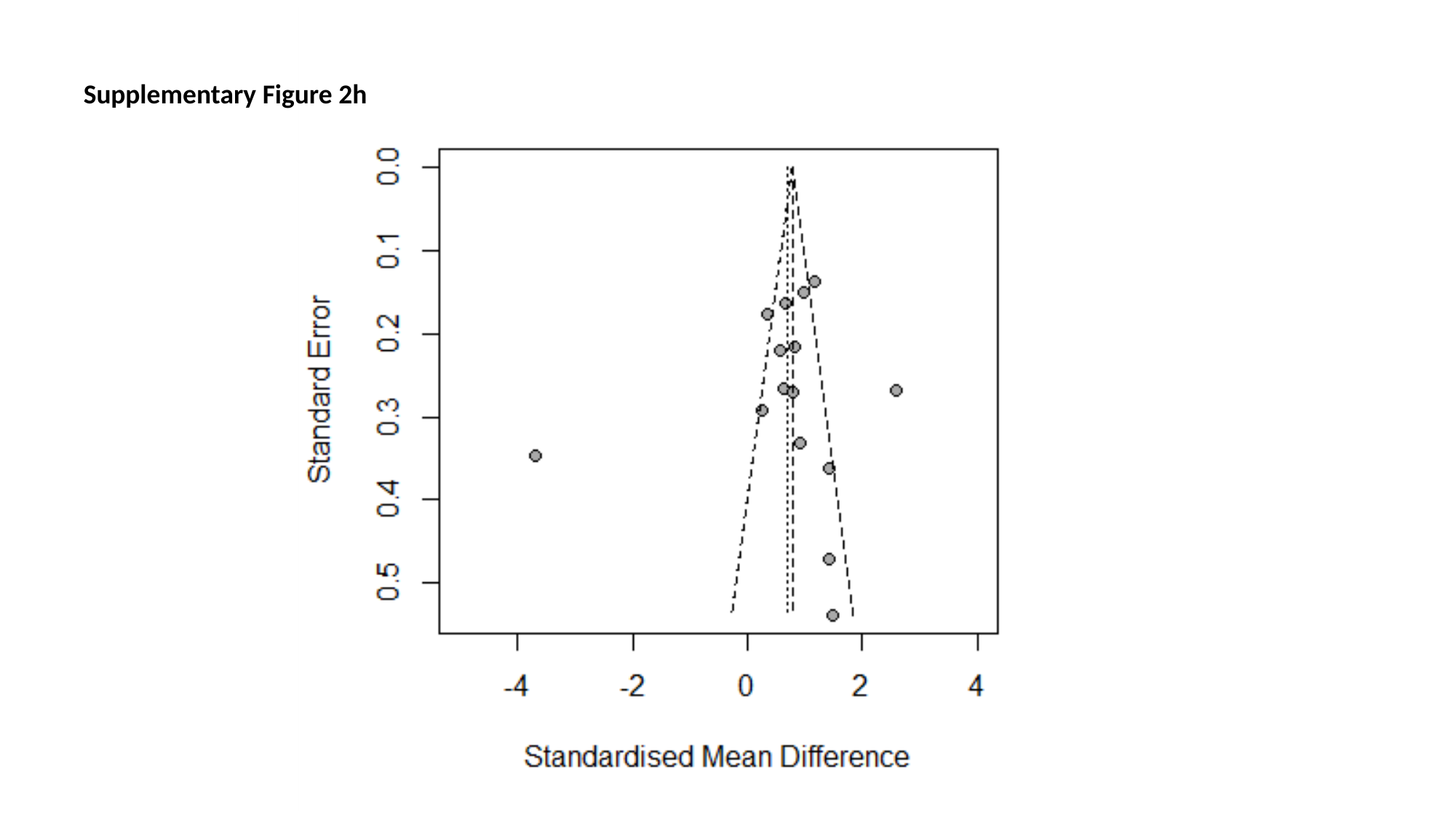

Supplementary Figure 2h

## Slide 9
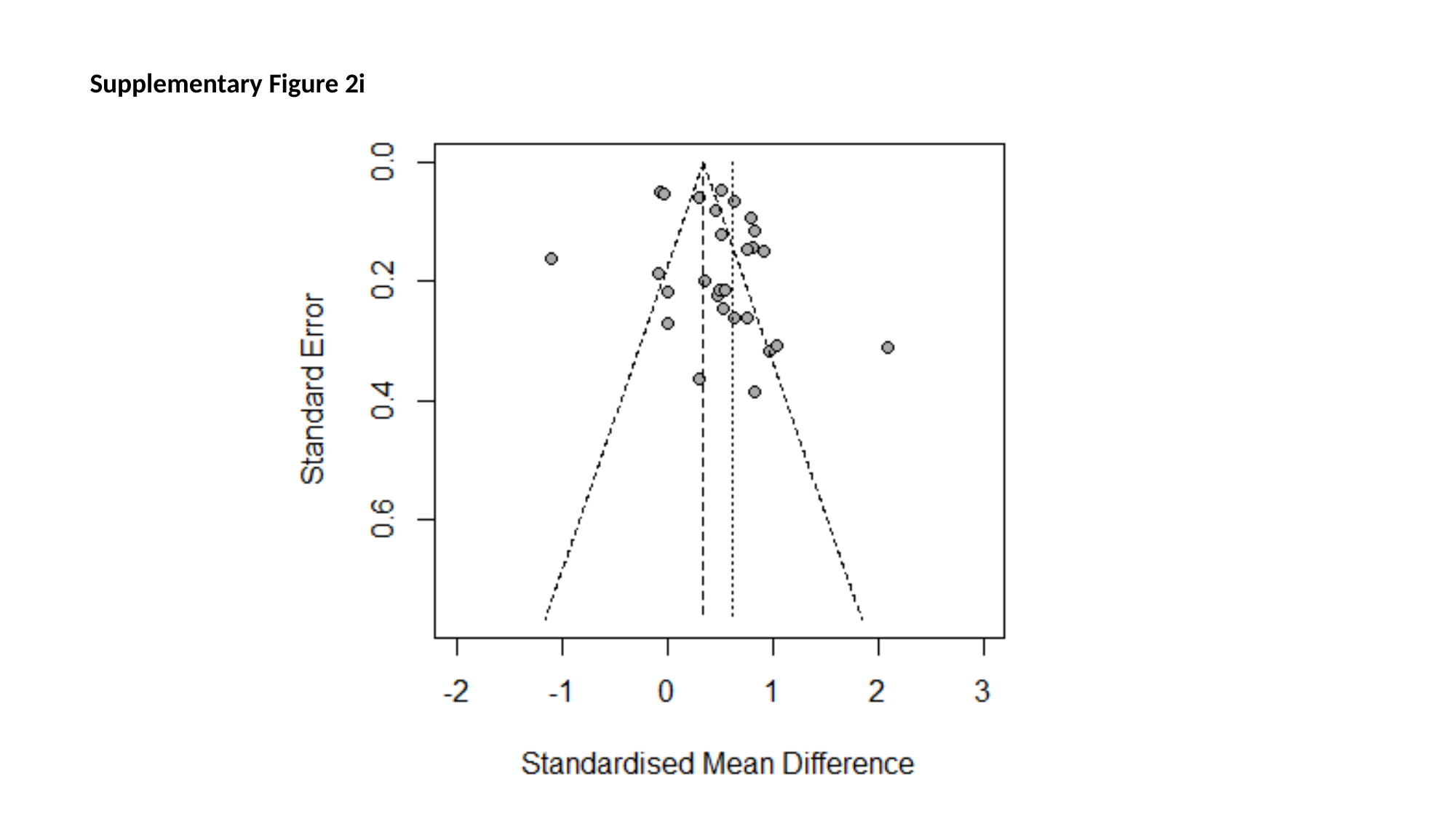

Supplementary Figure 2i

## Slide 10
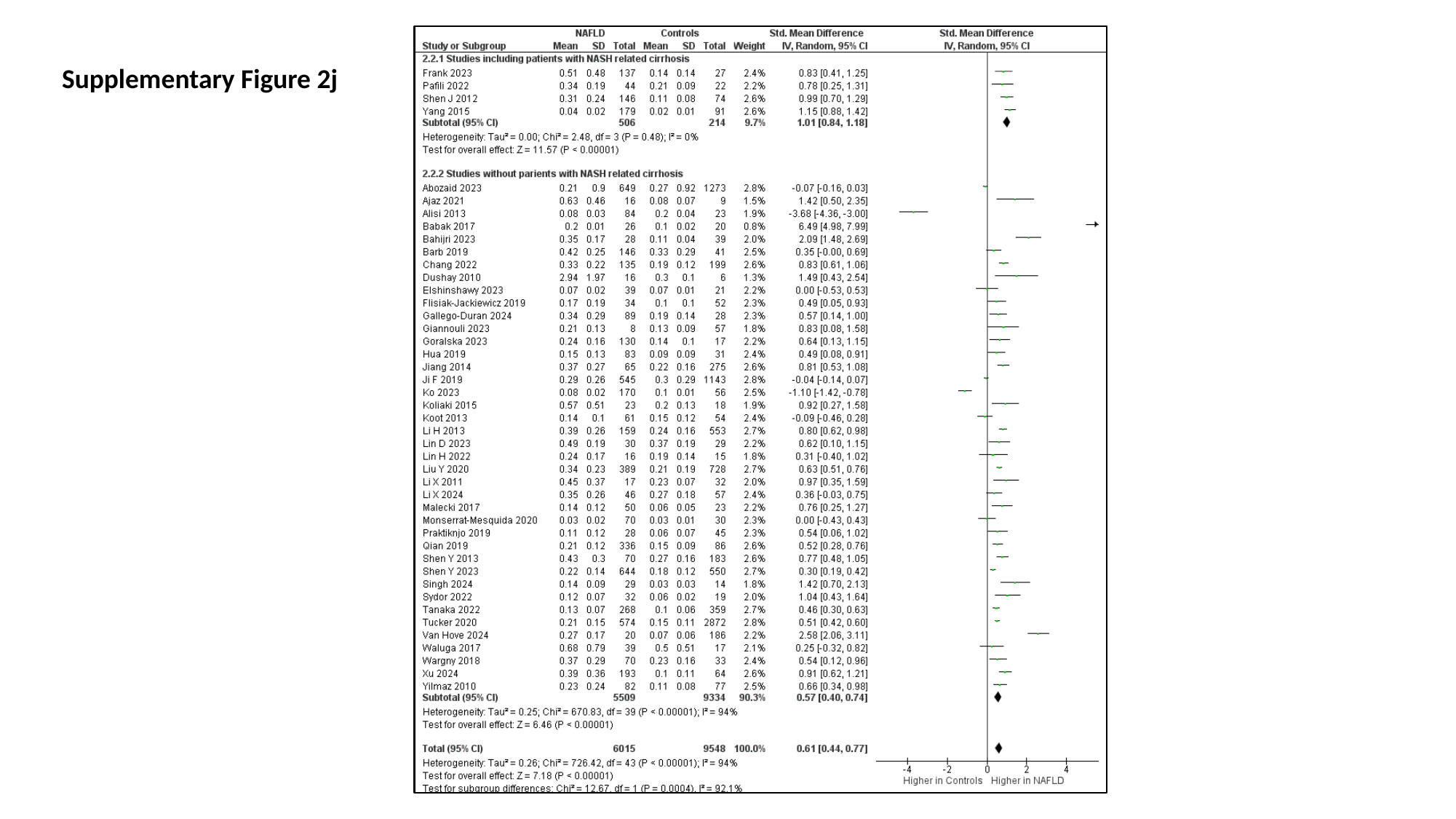

Supplementary Figure 2j

## Slide 11
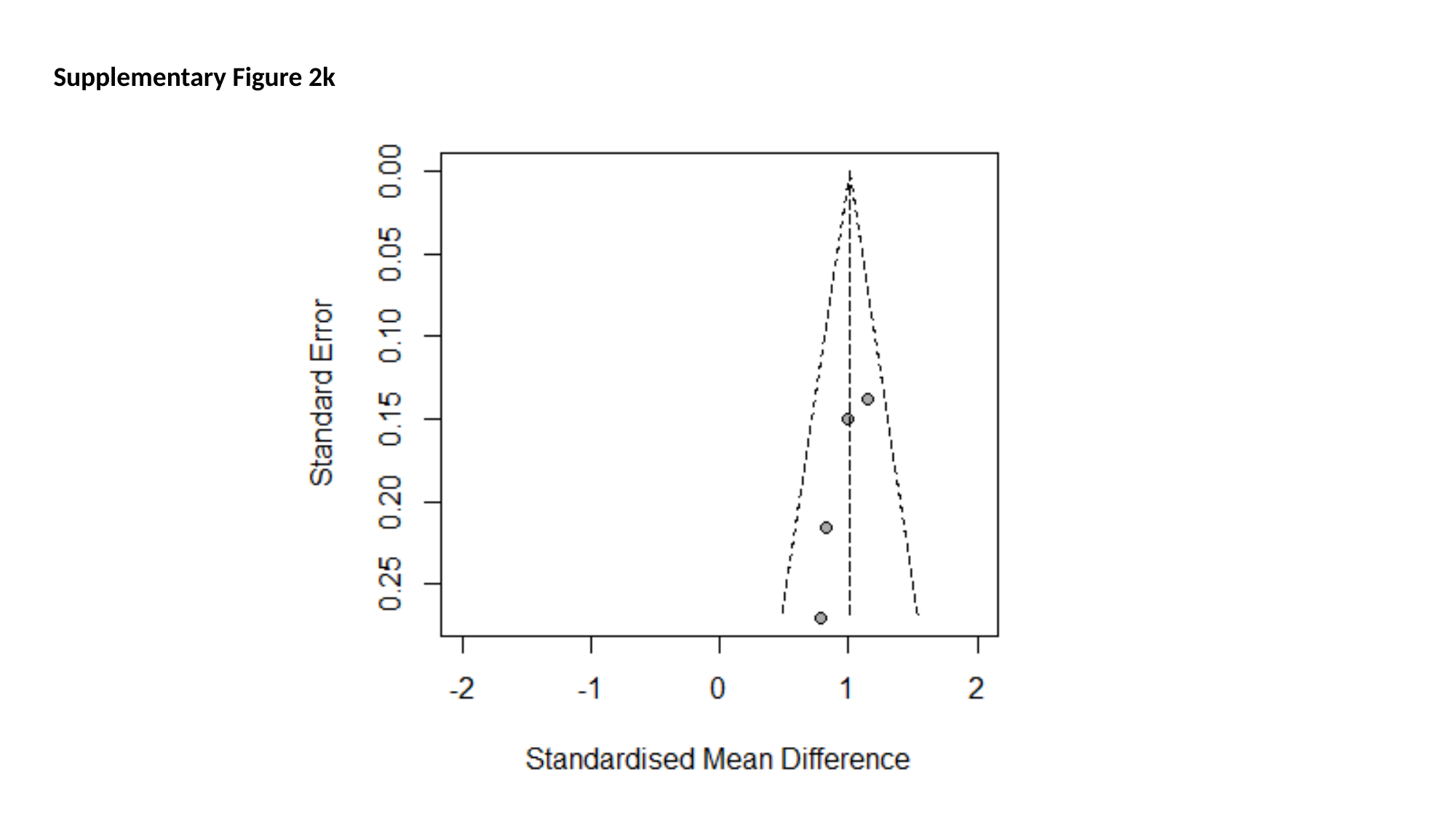

Supplementary Figure 2k

## Slide 12
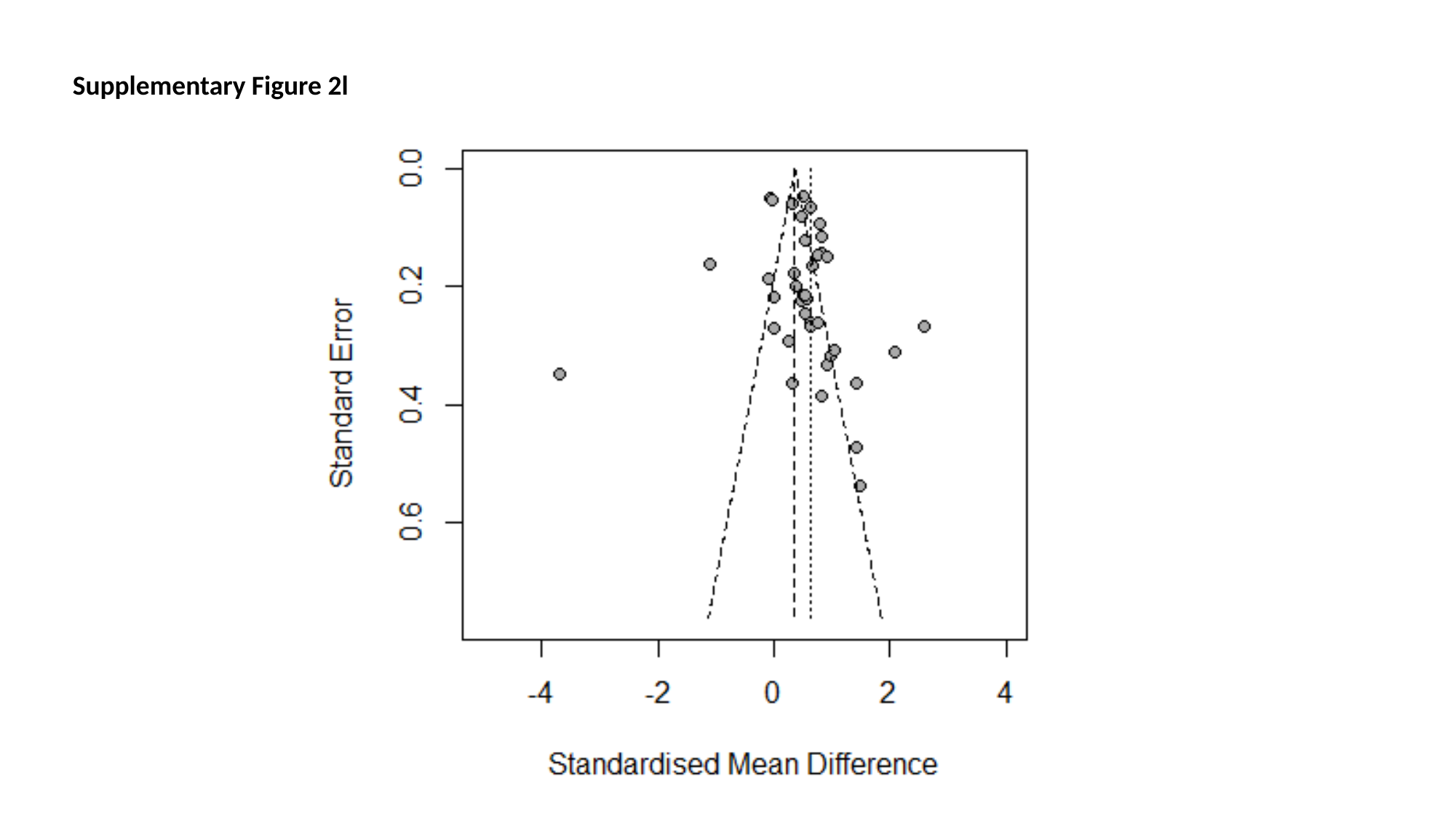

Supplementary Figure 2l
