# Supplementary material for: Circulating Fibroblast Growth Factor-21 in Patients with Nonalcoholic Fatty Liver Disease: A Systematic Review and Meta-Analysis
Source: Curr Obes Rep. 2025 Jun 4;14(1):51. doi: 10.1007/s13679-025-00643-x (PMC12137391; doi:10.1007/s13679-025-00643-x)
Supplement: Supplementary file 3 — (PPTX 191 KB) [file 13679_2025_643_MOESM3_ESM.pptx]

## Slide 1
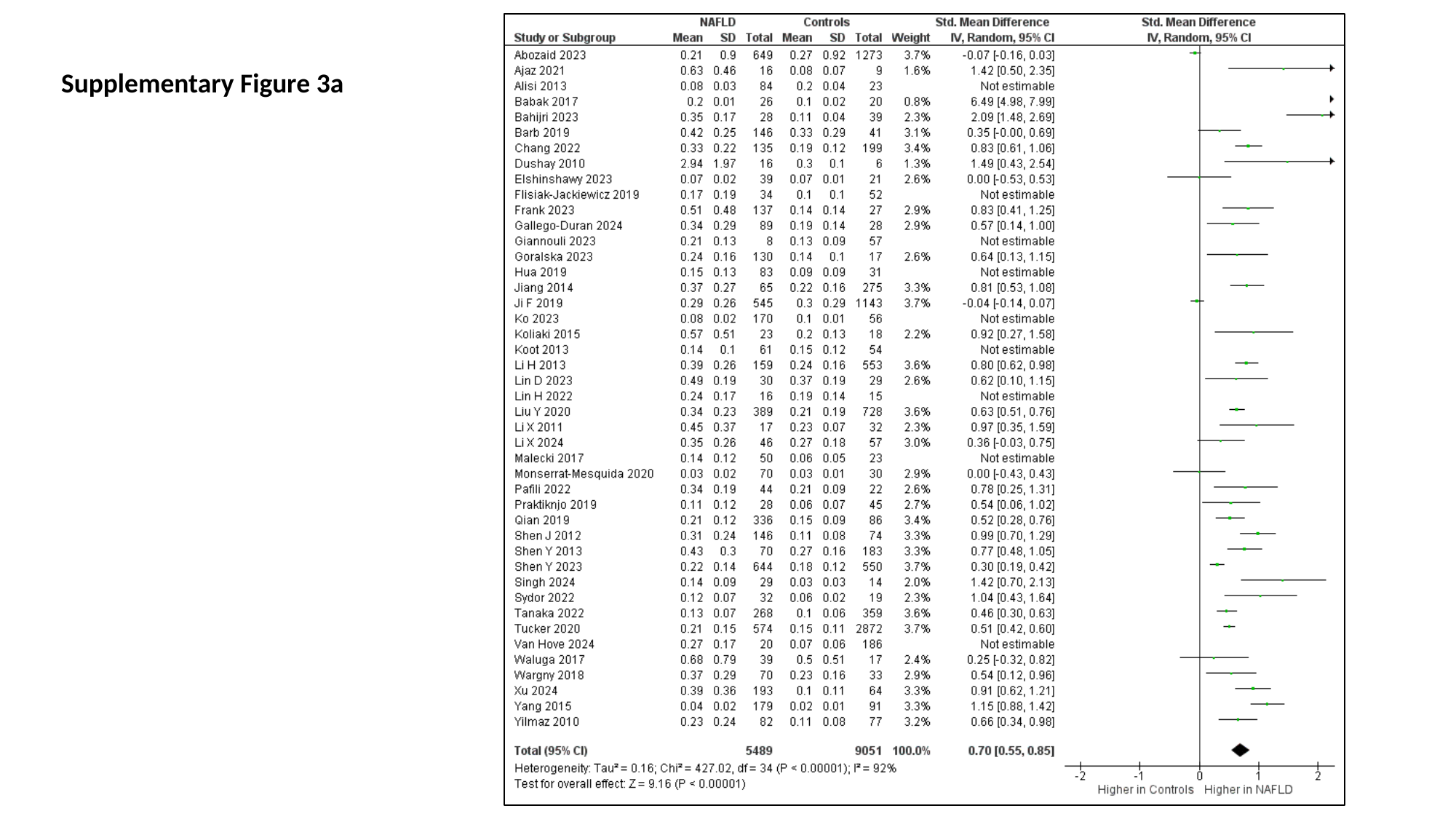

Supplementary Figure 3a

## Slide 2
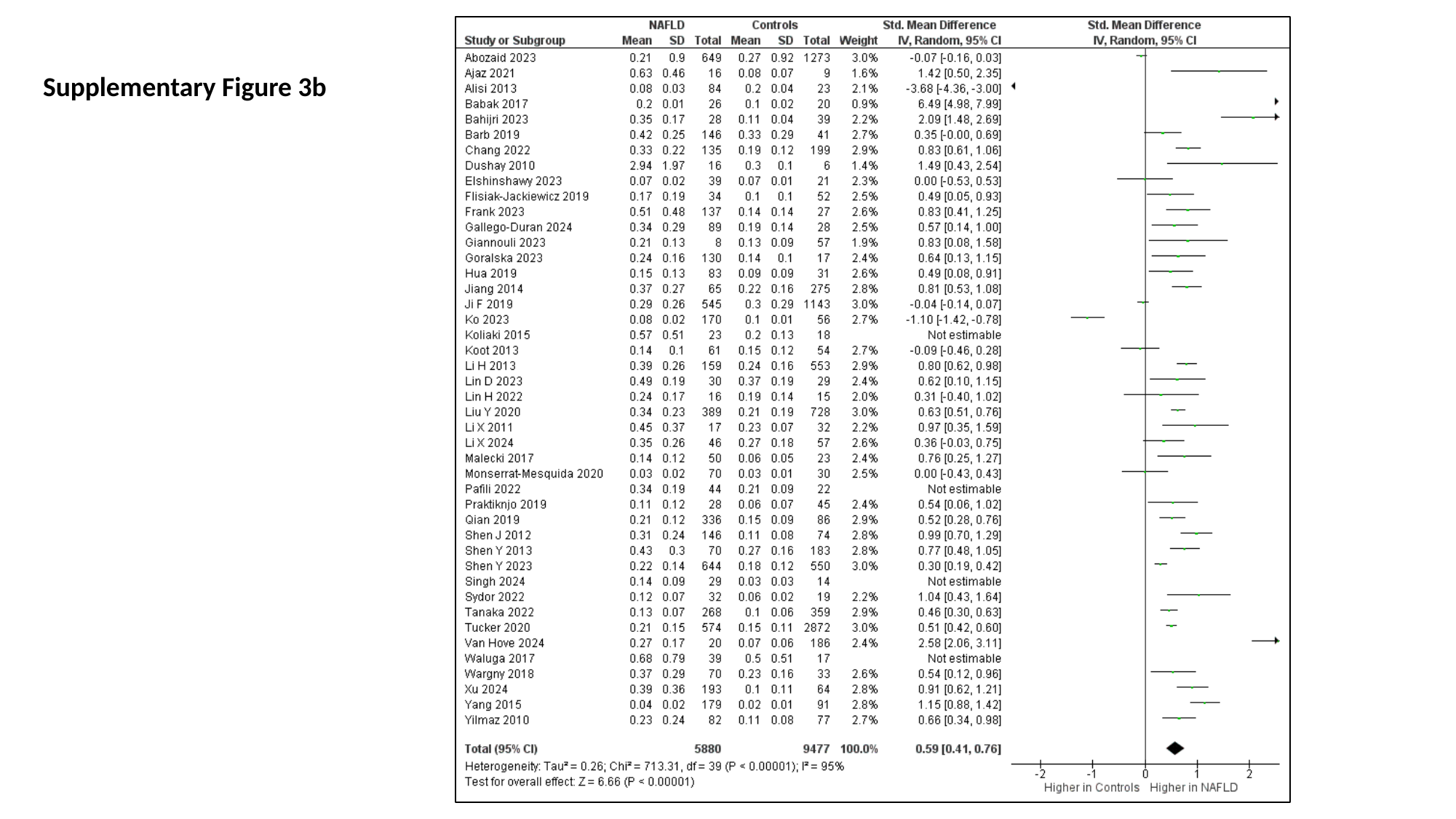

Supplementary Figure 3b

## Slide 3
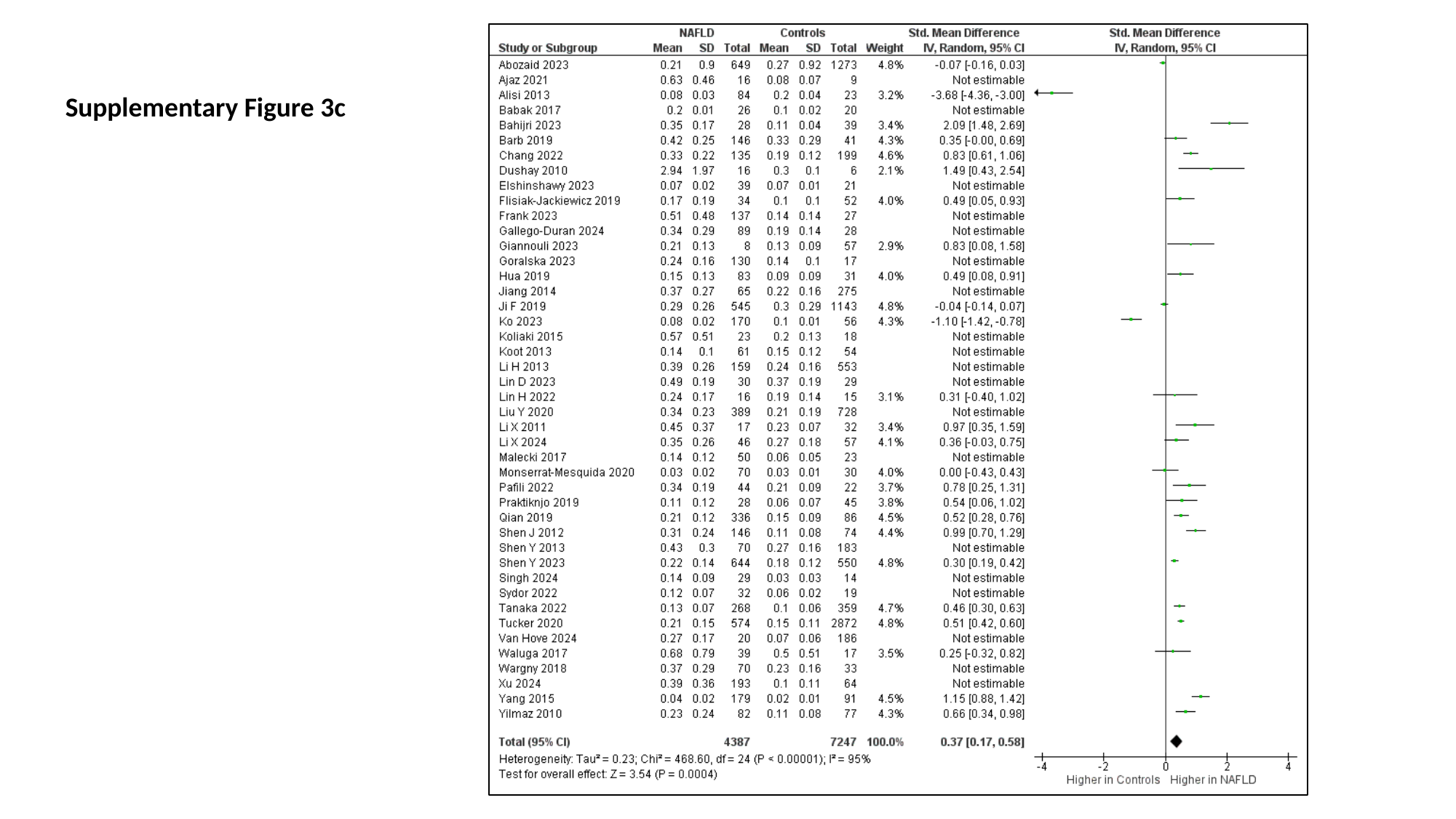

Supplementary Figure 3c

## Slide 4
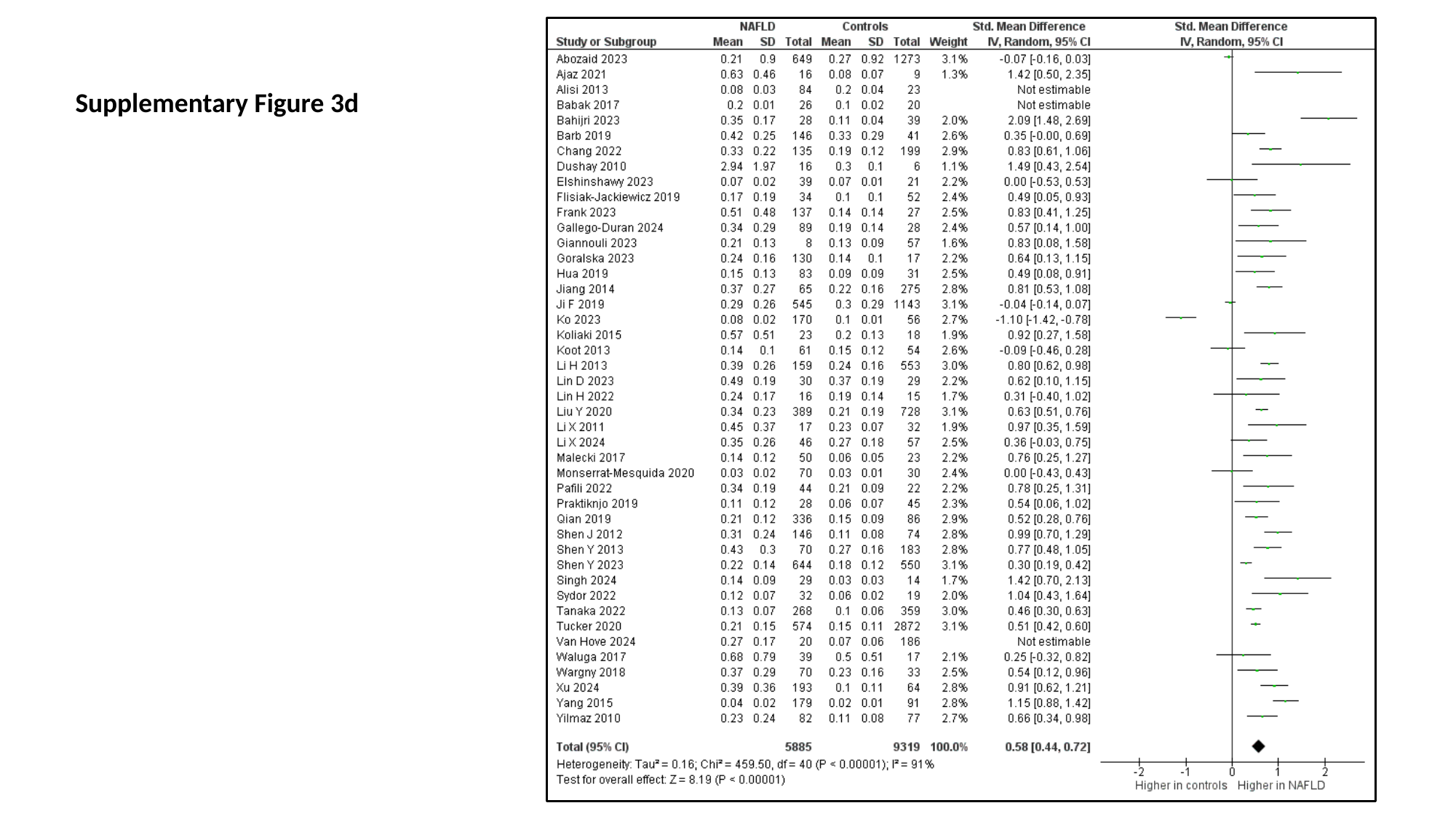

Supplementary Figure 3d

## Slide 5
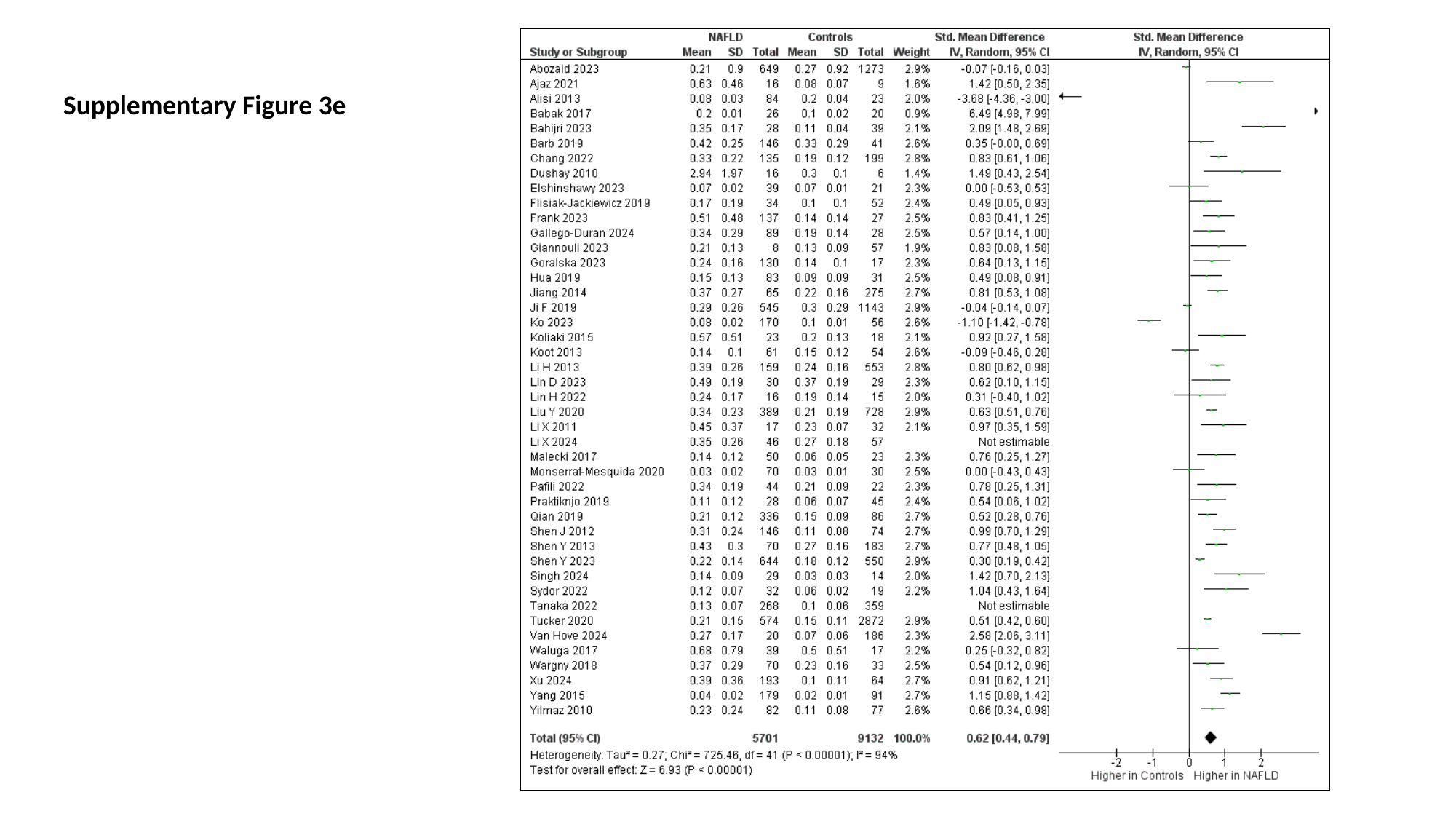

Supplementary Figure 3e
