# Supplementary material for: Circulating Fibroblast Growth Factor-21 in Patients with Nonalcoholic Fatty Liver Disease: A Systematic Review and Meta-Analysis
Source: Curr Obes Rep. 2025 Jun 4;14(1):51. doi: 10.1007/s13679-025-00643-x (PMC12137391; doi:10.1007/s13679-025-00643-x)
Supplement: Supplementary file 6 — (DOCX 107 KB) [file 13679_2025_643_MOESM6_ESM.docx]

| **Supplementary Table 1**. Main anthropometric and laboratory characteristics per group of each study included in the systematic review and meta-analysis. | | | | | | | | | | | | |
| --- | --- | --- | --- | --- | --- | --- | --- | --- | --- | --- | --- | --- |
| **First author, Year, Origin^†^** | **Group** | **Participants**  **(men, N)** | **Age (years)** | **BMI (kg/m^2^)** | **Waist circumference (cm)** | **T2DM (N)** | **FGF-21 (ng/ml)** | **AST (IU/L)** | **ALT (IU/L)** | **GGT (IU/L)** | **HOMA-IR** | **NASH-related cirrhosis (Ν)** |
| Abozaid, 2023, Netherlands [13] | Controls | 1273 (505) | 56.09 ± 6.03 | 26.57 ± 3.97 | NA | NA | 0.27 ± 0.92 | NA | NA | NA | NA | NA |
|  | NAFLD | 649 (293) | 56.55 ± 5.84 | 29.79 ± 4.49 | NA | NA | 0.21 ± 0.90 | NA | NA | NA | NA | NA |
| Ajaz, 2021, United Kingdom [14] | Controls | 9 (NA) | NA | NA | NA | NA | 0.08 ± 0.07 | NA | NA | NA | NA | 0 |
|  | NAFLD | 16 (NA) | NA | NA | NA | NA | 0.63 ± 0.46 | NA | NA | NA | NA | NA |
| Alisi, 2013, Italy [25] | Controls | 23 (14) | 10.00 ± 8.69 | 25.63 + 2.37 | NA | NA | 0.20 ± 0.04 | 28.33 ± 3.16 | 30.00 ± 6.32 | 20.67 ± 3.16 | NA | 0 |
|  | NAFLD | 84 (52) | 10.00 ± 1.51 | 24.87 + 3.92 | NA | NA | 0.08 ± 0.03 | 52.00 ± 18.86 | 69.33 ± 1.68 | 25.00 ± 12.82 | NA | 0 |
| Babak, 2017, Ukraine [36] | Controls | 20 (NA) | NA | NA | NA | NA | 0.10 ± 0.02 | 25.60 ± 1.74 | 22.70 ± 1.70 | NA | NA | NA |
|  | NAFLD | 26 (14) | 46.36 ± 2.19 | 27.53 ± 0.00 | NA | NA | 0.20 ± 0.01 | 42.16 ± 1.32 | 51.50 ± 1.29 | NA | NA | NA |
| Bahijri, 2023, Saudi Arabia [47] | Controls | 39 (16) | 57.00 ± 10.60 | 39.14 ± 36.35 | 101.65 ± 13.42 | 39 | 0.11 ± 0.04 | 15.82 ± 4.99 | 16.15 ± 7.20 | 32.40 ± 16.70 | 6.18 ± 5.27 | NA |
|  | NAFLD | 28 (6) | 57.40 ± 11.90 | 42.87 ± 8.58 | 114.13 ± 12.31 | 28 | 0.35 ± 0.17 | 21.32 ± 11.20 | 22.75 ± 16.59 | 50.30 ± 62.80 | 10.40 ± 10.40 | NA |
| Barb, 2019, USA [52] | Controls | 41 (28) | 55.00 ± 11.00 | 32.10 ± 3.80 | NA | 21 | 0.33 ± 0.29 | 29.00 ± 17.00 | 33.00 ± 27.00 | NA | NA | NA |
|  | NAFLD | 146 (107) | 38.86 ± 9.70 | 34.44 ± 4.67 | NA | 79 | 0.42 ± 0.25 | 44.23 ± 25.24 | 63.10 ± 36.14 | NA | NA | NA |
| Chang, 2022, South Korea [53] | Controls | 199 (63) | 66.33 ± 8.21 | 24.07 ± 2.61 | 81.67 ± 8.96 | 35 | 0.19 ± 0.12 | 22.00 ± 4.48 | 17.00 ± 5.97 | 18.67 ± 8.96 | 1.67 ± 1.27 | NA |
|  | NAFLD | 135 (56) | 65.67 ± 7.49 | 26.70 ± 2.62 | 89.33 ± 8.24 | 37 | 0.33 ± 0.22 | 24.00 ± 6.74 | 22.00 ± 8.24 | 29.33 ± 21.73 | 3.27 ± 2.70 | NA |
| Dushay, 2010, USA and Spain [54] | Controls | 6 (3) | NA | NA | NA | NA | 0.30 ± 0.10 | NA | NA | NA | NA | NA |
|  | NAFLD | 16 (NA) | NA | 48.20 | NA | NA | 2.94 ± 1.97 | NA | NA | NA | NA | NA |
| Elshinshawy, 2023, Egypt [55] | Controls | 21 (NA) | 33.80 ± 11.29 | 26.85 ± 4.92 | 84.97 ± 11.73 | 0 | 0.07 ± 0.01 | 23.09 ± 9.95 | 20.31 ± 10.42 | 23.83 ± 9.93 | 2.01 ± 1.68 | NA |
|  | NAFLD | 39 (NA) | 36.41 ± 9.03 | 34.47 ± 5.09 | 101.13 ± 9.90 | 0 | 0.07 ± 0.02 | 25.20 ± 16.78 | 24.01 ± 17.06 | 33.56 ± 24.78 | 2.99 ± 2.23 | NA |
| Flisiak-Jackiewicz, 2019, Poland [56] | Controls | 52 (NA) | 12.00 ± 3.05 | 27.62 ± 3.89 | 93.33 ± 12.20 | 0 | 0.10 ± 0.10 | NA | 26.33 ± 13.34 | 18.33 ± 6.86 | 3.04 ± 1.41 | NA |
|  | NAFLD | 34 (NA) | 14.00 ± 3.10 | 29.37 ± 5.19 | 100.00 ± 10.06 | 0 | 0.17 ± 0.19 | NA | 71.67 ± 38.70 | 32.50 ± 17.80 | 3.80 ± 1.53 | NA |
| Franck, 2023, Germany [15] | Controls | 27 (NA) | NA | NA | NA | NA | 0.14 ± 0.14 | NA | NA | NA | NA | 0 |
|  | NAFLD | 137 (78) | 46.69 ± 1.56 | 29.91 ± 1.32 | NA | 30 | 0.51 ± 0.48 | NA | 86.09 ± 7.46 | NA | NA | 7 |
| Gallego-Duran, 2024, Spain [16] | Controls | 28 (NA) | NA | NA | NA | NA | 0.19 ± 0.14 | NA | NA | NA | NA | NA |
|  | NAFLD | 89 (NA) | NA | NA | NA | NA | 0.34 ± 0.29 | NA | NA | NA | NA | NA |
| Giannouli, 2023, Greece [17] | Controls | 57 (NA) | NA | NA | NA | NA | 0.13 ± 0.09 | NA | NA | NA | NA | NA |
|  | NAFLD | 8 (NA) | NA | NA | NA | NA | 0.21 ± 0.13 | NA | NA | NA | NA | NA |
| Goralska, 2023, Poland [18] | Controls | 17 (NA) | NA | NA | NA | NA | 0.14 ± 0.10 | NA | NA | NA | NA | NA |
|  | NAFLD | 130 (NA) | NA | NA | NA | NA | 0.24 ± 0.16 | NA | NA | NA | NA | NA |
| Hua, 2019, Taiwan [19] | Controls | 31 (13) | 13.74 ± 3.60 | 24.54 ± 3.54 | 80.76 ± 11.91 | NA | 0.09 ± 0.09 | 18.04 ± 4.78 | 17.41 ± 11.03 | 15.17 ± 4.72 | NA | NA |
|  | NAFLD | 83 (46) | 12.78 ± 3.27 | 27.30 ± 3.53 | 88.40 ± 14.83 | NA | 0.15 ± 0.13 | 33.77 ± 23.32 | 50.18 ± 15.94 | 24.78 ± 12.63 | NA | NA |
| Ji, 2019, China [20] | Controls | 1143 (487) | 42.22 ± 12.52 | 23.99 ± 3.33 | NA | NA | 0.30 ± 0.29 | 21.25 ± 9.72 | 23.67 ± 25.52 | 25.46 ± 27.33 | NA | NA |
|  | NAFLD | 545 (218) | 42.39 ± 12.60 | 24.11 ± 3.51 | NA | NA | 0.29 ± 0.26 | 25.18 ± 14.51 | 23.78 ± 17.59 | 24.95 ± 20.91 | NA | NA |
| Jiang, 2014, China [21] | Controls | 275 (101) | 43.74 ± 12.34 | 22.30 ± 2.46 | NA | 0 | 0.22 ± 0.16 | 19.33 ± 5.22 | 14.67 ± 5.96 | 17.67 ± 8.20 | NA | NA |
|  | NAFLD | 65 (34) | 47.58 ± 9.30 | 27.33 ± 2.35 | NA | 0 | 0.37 ± 0.27 | 22.67 ± 7.58 | 24.00 ± 15.16 | 26.67 ± 15.16 | NA | NA |
| Ko, 2023, South Korea [22] | Controls | 56 (25) | 11.32 ± 1.80 | 25.98 ± 0.42 | 79.26 ± 1.17 | NA | 0.10 ± 0.01 | 20.01 ± 3.14 | 15.78 ± 5.13 | 16.91 ± 2.65 | 4.25 ± 0.42 | NA |
|  | NAFLD | 170 (130) | 11.39 ± 1.59 | 29.17 ± 0.82 | 88.63 ± 2.09 | NA | 0.08 ± 0.02 | 34.91 ± 11.72 | 48.13 ± 21.34 | 30.01 ± 7.99 | 6.64 ± 1.20 | NA |
| Koliaki, 2015, Germany [23] | Controls | 18 (3) | 39.00 ± 3.00 | 48.30 ± 1.90 | NA | NA | 0.20 ± 0.13 | 31.00 ± 4.00 | 31.00 ± 13.00 | NA | NA | NA |
|  | NAFLD | 23 (5) | 44.04 ± 5.54 | 51.75 ± 3.49 | NA | 3 | 0.57 ± 0.51 | 39.91 ± 15.47 | 45.09 ± 10.45 | NA | NA | NA |
| Koot, 2013, Netherlands [24] | Controls | 54 (NA) | NA | NA | NA | 0 | 0.15 ± 0.12 | NA | NA | NA | NA | NA |
|  | NAFLD | 61 (NA) | 37.37 ± 36.63 | NA | NA | 0 | 0.14 ± 0.10 | NA | NA | NA | NA | NA |
| Li H, 2013, China [26] | Controls | 553 (200) | 45.83 ± 13.12 | 22.54 ± 2.85 | 73.30 ± 7.98 | NA | 0.24 ± 0.16 | 19.33 ± 5.20 | 14.67 ± 5.95 | 17.33 ± 7.43 | NA | NA |
|  | NAFLD | 159 (78) | 48.74 ± 11.11 | 27.25 ± 2.47 | 86.51 ± 6.62 | NA | 0.39 ± 0.26 | 23.67 ± 7.48 | 27.67 ± 19.83 | 32.50 ± 22.01 | NA | NA |
| Li X, 2011, China [27] | Controls | 32 (18) | 63.91 ± 11.56 | 24.41 ± 3.87 | NA | 32 | 0.23 ± 0.07 | 31.60 ± 20.41 | 26.20 ± 10.61 | 34.70 ± 14.20 | 4.63 ± 2.08 | NA |
|  | NAFLD | 17 (8) | 63.00 ± 12.12 | 25.40 ± 4.83 | NA | 17 | 0.45 ± 0.37 | 29.70 ± 12.96 | 26.00 ± 7.69 | 60.73 ± 28.62 | 6.06 ± 2.64 | NA |
| Li X, 2024, China [28] | Controls | 57 (27) | 47.68 ± 12.10 | 24.46 ± 3.81 | 84.00 ± 11.07 | 0 | 0.27 ± 0.18 | NA | NA | NA | 2.02 ± 1.20 | NA |
|  | NAFLD | 46 (24) | 52.02 ± 10.31 | 29.78 ± 4.13 | 97.57 ± 10.74 | 0 | 0.35 ± 0.26 | NA | NA | NA | 3.25 ± 1.07 | NA |
| Lin D, 2023, China [29] | Controls | 29 (23) | 33.30 ± 1.90 | 23.00 ± 4.90 | 76.50 ± 11.70 | NA | 0.37 ± 0.19 | NA | NA | NA | NA | NA |
|  | NAFLD | 30 (25) | 34.80 ± 14.10 | 29.90 ± 4.30 | 97.80 ± 7.90 | NA | 0.49 ± 0.19 | NA | NA | NA | NA | 0 |
| Lin Η, 2022, USA [30] | Controls | 15 (8) | 14.05 ± 1.92 | 35.96 ± 4.90 | NA | 0 | 0.19 ± 0.14 | 26.53 ± 6.25 | 34.00 ± 16.87 | 23.26 ± 9.37 | 4.90 ± 2.01 | NA |
|  | NAFLD | 16 (8) | 14.10 ± 2.10 | 36.20 ± 4.70 | NA | 0 | 0.24 ± 0.17 | 33.00 ± 13.00 | 47.00 ± 29.00 | 29.00 ± 13.00 | 12.40 ± 8.50 | NA |
| Liu, 2020, China [31] | Controls | 728 (289) | 40.33 ± 14.11 | 22.73 ± 2.67 | NA | NA | 0.21 ± 0.19 | 20.00 ± 4.46 | 17.33 ± 8.91 | 16.75 ± 7.24 | NA | NA |
|  | NAFLD | 389 (202) | 46.00 ± 14.88 | 26.69 ± 3.04 | NA | NA | 0.34 ± 0.23 | 22.67 ± 5.95 | 25.67 ± 16.37 | 28.00 ± 17.11 | NA | NA |
| Małecki, 2017, Poland [32] | Controls | 23 (13) | 10.90 ± 3.91 | 18.51 ± 2.31 | NA | NA | 0.06 ± 0.05 | 27.61 ± 11.17 | 17.13 ± 6.65 | NA | NA | NA |
|  | NAFLD | 50 (36) | 10.80 ± 3.07 | 29.04 ± 6.06 | NA | NA | 0.14 ± 0.12 | 45.78 ± 18.35 | 61.64 ± 17.71 | NA | NA | NA |
| Monserrat-Mesquida, 2020, Spain [33] | Controls | 30 (14) | 52.50 ± 1.10 | 32.40 ± 0.70 | NA | NA | 0.03 ± 0.01 | 22.60 ± 1.80 | 27.80 ± 2.20 | 48.50 ± 5.90 | NA | NA |
|  | NAFLD | 70 (39) | 52.60 ± 1.46 | 33.65 ± 0.92 | NA | NA | 0.03 ± 0.02 | 26.95 ± 2.62 | 39.20 ± 7.13 | 48.85 ± 5.30 | NA | NA |
| Pafili, 2022, Germany [34] | Controls | 22 (2) | 37.00 ± 11.09 | 52.67 ± 10.30 | 132.00 ± 12.68 | 2 | 0.21 ± 0.09 | NA | 24.67 ± 11.89 | NA | NA | 0 |
|  | NAFLD | 44 (8) | 41.17 ± 11.74 | 50.67 ± 5.55 | 136.33 ± 12.15 | 14 | 0.34 ± 0.19 | NA | 36.16 ± 19.02 | NA | NA | 1 |
| Praktiknjo, 2019, Germany [35] | Controls | 45 (NA) | NA | NA | NA | NA | 0.06 ± 0.07 | NA | NA | NA | NA | 0 |
|  | NAFLD | 28 (NA) | NA | NA | NA | NA | 0.11 ± 0.12 | NA | NA | NA | NA | 0 |
| Qian, 2019, China [37] | Controls | 86 (47) | 46.33 ± 18.10 | 25.04 ± 3.31 | 88.06 ± 8.04 | NA | 0.15 ± 0.09 | 16.75 ± 3.20 | 14.58 ± 6.22 | 20.92 ± 11.50 | 2.49 ± 1.33 | NA |
|  | NAFLD | 336 (235) | 41.25 ± 12.47 | 27.54 ± 3.45 | 95.38 ± 8.84 | NA | 0.21 ± 0.12 | 21.58 ± 7.26 | 26.00 ± 14.15 | 33.67 ± 17.12 | 3.17 ± 1.18 | NA |
| Shen J, 2012, China [38] | Controls | 74 (40) | 47.40 ± 10.30 | 22.50 ± 2.70 | 81.00 ± 8.00 | 1 | 0.11 ± 0.08 | NA | 28.00 ± 26.00 | NA | NA | 0 |
|  | NAFLD | 146 (81) | 48.10 ± 9.70 | 27.40 ± 3.90 | 94.00 ± 11.00 | 70 | 0.31 ± 0.24 | NA | 71.00 ± 42.00 | NA | NA | 11 |
| Shen Y, 2023, China [39] | Controls | 550 (205) | 59.00 ± 5.95 | 22.57 ± 2.53 | NA | NA | 0.18 ± 0.12 | 15.67 ± 5.95 | 20.67 ± 4.46 | NA | 1.61 ± 0.81 | NA |
|  | NAFLD | 644 (235) | 60.00 ± 5.94 | 25.20 ± 2.90 | NA | NA | 0.22 ± 0.14 | 20.33 ± 8.92 | 21.67 ± 5.94 | NA | 2.58 ± 1.41 | NA |
| Shen Y, 2013, China [40] | Controls | 183 (121) | 67.20 ± 10.00 | 23.90 ± 3.10 | 88.40 ± 9.30 | NA | 0.27 ± 0.16 | NA | NA | NA | 3.83 ± 2.17 | NA |
|  | NAFLD | 70 (27) | 63.90 ± 9.90 | 26.50 ± 3.70 | 95.40 ± 9.30 | NA | 0.43 ± 0.30 | NA | NA | NA | 5.63 ± 2.65 | NA |
| Singh, 2024, USA [41] | Controls | 14 (NA) | NA | NA | NA | 0 | 0.03 ± 0.03 | NA | NA | NA | NA | NA |
|  | NAFLD | 29 (NA) | NA | NA | NA | NA | 0.14 ± 0.09 | NA | NA | NA | NA | NA |
| Sydor, 2022, Germany [42] | Controls | 19 (17) | 23.50 ± 2.70 | 23.00 ± 2.60 | NA | NA | 0.06 ± 0.02 | 26.00 ± 13.70 | 25.40 ± 15.00 | 18.20 ± 8.00 | NA | NA |
|  | NAFLD | 32 (20) | 53.30 ± 14.00 | 30.90 ± 6.50 | NA | NA | 0.12 ± 0.07 | 56.10 ± 43.70 | 66.40 ± 56.60 | 132.60 ± 120.30 | NA | NA |
| Tanaka, 2022, Japan [43] | Controls | 359 (176) | 64.00 ± 16.00 | 21.30 ± 2.40 | 79.40 ± 8.00 | NA | 0.10 ± 0.06 | 22.33 ± 5.21 | 17.00 ± 6.70 | 20.33 ± 8.93 | 1.88 ± 1.80 | NA |
|  | NAFLD | 268 (116) | 66.00 ± 14.00 | 26.30 ± 3.40 | 94.00 ± 8.50 | NA | 0.13 ± 0.07 | 24.00 ± 5.96 | 22.33 ± 9.69 | 30.33 ± 18.63 | 3.05 ± 2.91 | NA |
| Tucker, 2020, USA [44] | Controls | 2872 (1304) | 63.80 ± 10.50 | 28.00 ± 5.20 | 97.30 ± 13.60 | NA | 0.15 ± 0.11 | NA | NA | 8.13 ± 5.56 | 0.91 ± 0.43 | NA |
|  | NAFLD | 574 (271) | 61.60 ± 9.60 | 31.20 ± 5.30 | 106.10 ± 13.10 | NA | 0.21 ± 0.15 | NA | NA | 12.63 ± 8.03 | 1.52 ± 0.74 | NA |
| Van Hove, 2024, USA [45] | Controls | 186 (100) | 7.11 ± 8.97 | NA | NA | NA | 0.07 ± 0.06 | NA | NA | NA | NA | NA |
|  | NAFLD | 20 (9) | 12.78 ± 3.06 | 31.23 ± 4.87 | NA | NA | 0.27 ± 0.17 | 136.00 ± 97.34 | NA | 63.67 ± 43.88 | NA | NA |
| Waluga, 2017, Poland [46] | Controls | 17 (NA) | NA | NA | NA | NA | 0.50 ± 0.51 | NA | NA | NA | NA | 0 |
|  | NAFLD | 39 (NA) | NA | NA | NA | NA | 0.68 ± 0.79 | NA | NA | NA | NA | 0 |
| Wargny, 2018, France [48] | Controls | 33 (21) | NA | 28.80 ± 5.30 | NA | 5 | 0.23 ± 0.16 | NA | 31.40 ± 21.50 | 56.20 ± 69.50 | NA | NA |
|  | NAFLD | 70 (49) | NA | 32.50 ± 5.20 | NA | 16 | 0.37 ± 0.29 | NA | 46.60 ± 29.60 | 78.60 ± 101.50 | NA | NA |
| Xu, 2024, China [49] | Controls | 64 (32) | 47.17 ± 12.13 | 22.51 ± 2.19 | NA | NA | 0.10 ± 0.11 | 18.70 ± 5.20 | 14.94 ± 7.11 | NA | NA | NA |
|  | NAFLD | 193 (110) | 53.00 ± 7.47 | 26.24 ± 3.02 | NA | NA | 0.39 ± 0.36 | 21.47 ± 5.60 | 22.27 ± 11.65 | NA | NA | NA |
| Yang, 2015, China [50] | Controls | 91 (42) | 29.40 ± 7.00 | 19.80 ± 2.10 | NA | NA | 0.02 ± 0.01 | 29.60 ± 5.90 | 27.20 ± 10.30 | 36.30 ± 17.60 | NA | 0 |
|  | NAFLD | 179 (86) | 30.45 ± 12.71 | 27.21 ± 3.63 | NA | NA | 0.04 ± 0.02 | 60.36 ± 31.92 | 116.02 ± 49.50 | 106.60 ± 33.60 | NA | 7 |
| Yilmaz, 2010, Turkey [51] | Controls | 77 (37) | 47.00 ± 6.00 | 27.50 ± 4.40 | 89.00 ± 11.00 | 0 | 0.11 ± 0.08 | 24.00 ± 9.00 | 21.00 ± 13.00 | NA | 1.60 ± 1.66 | 0 |
|  | NAFLD | 82 (38) | 47.00 ± 8.00 | 31.00 ± 4.80 | 102.00 ± 12.00 | 22 | 0.23 ± 0.24 | 44.00 ± 18.00 | 65.00 ± 32.00 | NA | 3.60 ± 2.04 | 0 |
| **†**: Studies are sorted alphabetically according to the surname of the first author.  Abbreviations: ALT, alanine aminotransferase; AST, aspartate aminotransferase; BMI, body mass index; FGF-21, fibroblast growth factor-21; GGT, gamma-glutamyl transferase; HOMA-IR, homeostasis model assessment-insulin resistance; N, number; NA, not available; NASH, nonalcoholic steatohepatitis; NAFLD, nonalcoholic fatty liver disease; T2DM, type 2 diabetes mellitus | | | | | | | | | | | | |
