# Supplementary material for: Circulating Fibroblast Growth Factor-21 in Patients with Nonalcoholic Fatty Liver Disease: A Systematic Review and Meta-Analysis
Source: Curr Obes Rep. 2025 Jun 4;14(1):51. doi: 10.1007/s13679-025-00643-x (PMC12137391; doi:10.1007/s13679-025-00643-x)
Supplement: Supplementary file 7 — (DOCX 28.2 KB) [file 13679_2025_643_MOESM7_ESM.docx]

**Legends to supplementary figures**

**Supplementary Fig. 1** Funnel plot for the comparison of circulating FGF-21 between patients with NAFLD and controls (all studies)

**Supplementary Fig. 2:** Forest and funnel plots of subgroup analyses.

**Supplementary Fig. 2a** Forest plot for the comparison of circulating FGF-21 between patients with hepatic steatosis (NAFL) and controls

**Supplementary Fig. 2b** Funnel plot for the comparison of circulating FGF-21 between patients with hepatic steatosis (NAFL) and controls

**Supplementary Fig. 2c** Forest plot for the comparison of circulating FGF-21 between patients with NASH and controls

**Supplementary Fig. 2d** Funnel plot for the comparison of circulating FGF-21 levels between patients with NASH and controls

**Supplementary Fig. 2e** Forest plot for the comparison of circulating FGF-21 between patients with NASH and hepatic steatosis (NAFL)

**Supplementary Fig. 2f** Funnel plot for the comparison of circulating FGF-21 between patients with NASH and hepatic steatosis (NAFL)

**Supplementary Fig. 2g** Forest plot for the comparison of circulating FGF-21 between patients with NAFLD and controls, in subgroup analysis within studies with and without histological confirmation of NAFLD

**Supplementary Fig. 2h** Funnel plot for the comparison of circulating FGF-21 between patients with NAFLD and controls, in studies with histological confirmation of NAFLD

**Supplementary Fig. 2i** Funnel plot for the comparison of circulating FGF-21 between patients with NAFLD and controls, in studies without histological confirmation of NAFLD

**Supplementary Fig. 2j** Forest plot for the comparison of FGF-21 levels between patients with NAFLD and controls, in subgroup analysis within studies with and without the inclusion of patients with NASH-related cirrhosis

**Supplementary Fig. 2k** Funnel plot for the comparison of circulating FGF-21 between patients with NAFLD and controls, in studies with the inclusion of patients with NASH-related cirrhosis

**Supplementary Fig. 2l** Funnel plot for the comparison of circulating FGF-21 between patients with NAFLD and controls, in studies without the inclusion of patients NASH-related cirrhosis

**Supplementary Fig. 3**: Forest plot for the comparison of circulating FGF-21 levels in sensitivity analyses

**Supplementary Fig. 3a** Forest plot for the comparison of circulating FGF-21 between patients with NAFLD and controls, in sensitivity analysis after excluding studies with pediatric/adolescent populations

**Supplementary Fig. 3b** Forest plot for the comparison of circulating FGF-21 between patients with NAFLD and controls, in sensitivity analysis after excluding studies with morbidly obese populations subjected to bariatric surgery

**Supplementary Fig. 3c** Forest plot for the comparison of circulating FGF-21 between patients with NAFLD and controls, in sensitivity analysis after excluding studies with NOS score <7

**Supplementary Fig. 3d** Forest plot for the comparison of circulating FGF-21 between patients with NAFLD and controls, in sensitivity analysis after excluding studies with outliers of FGF-21 SMD

**Supplementary Fig. 3e** Forest plot for the comparison of circulating FGF-21 between patients with NAFLD and controls, in sensitivity analysis after excluding studies with the use of the definition of MAFLD for the diagnosis of the disease

**Supplementary Fig. 4:** Funnel plots of sensitivity analyses.

**Supplementary Fig. 4a** Funnel plot for the comparison of circulating FGF-21 between patients with NAFLD and controls, in sensitivity analysis after excluding studies with pediatric/adolescent populations

**Supplementary Fig. 4b** Funnel plot for the comparison of circulating FGF-21 between patients with NAFLD and controls, in sensitivity analysis after excluding studies with morbidly obese populations subjected to bariatric surgery

**Supplementary Fig. 4c** Funnel plot for the comparison of circulating FGF-21 between patients with NAFLD and controls, in sensitivity analysis after excluding studies with NOS score <7

**Supplementary Fig. 4d** Funnel plot for the comparison of circulating FGF-21 levels between patients with NAFLD and controls, in sensitivity analysis after excluding studies with outliers of FGF-21 SMD

**Supplementary Fig. 4e** Funnel plot for the comparison of FGF-21 between patients with NAFLD and controls, in sensitivity analysis after excluding studies with the use of the definition of MAFLD for the diagnosis of the disease

**Supplementary Fig. 5** Bubble plot of the association of the percentage of patients with T2DM with FGF-21 SMD between patients with NAFLD and controls
